# Positive Feedback Regulation between KLF5 and XPO1 Promotes Cell Cycle Progression of Basal like Breast Cancer

**DOI:** 10.1002/advs.202412096

**Published:** 2025-01-30

**Authors:** Yu Tang, Rui Liu, Jing Zhu, Qian He, Chenglong Pan, Zhongmei Zhou, Jian Sun, Fubing Li, Longlong Zhang, Yujie Shi, Jing Yao, Dewei Jiang, Ceshi Chen

**Affiliations:** ^1^ Yunnan Key Laboratory of Breast Cancer Precision Medicine, Yunnan Cancer Hospital The Third Affiliated Hospital of Kunming Medical University Peking University Cancer Hospital Yunnan Kunming 650118 China; ^2^ Yunnan Key Laboratory of Breast Cancer Precision Medicine Institute of Biomedical Engineering Kunming Medical University Kunming 650000 China; ^3^ Department of Pathology The First Affiliated Hospital of Kunming Medical University Kunming 650032 China; ^4^ School of Continuing Education Kunming Medical University Kunming 650021 China; ^5^ Department of Pathology Henan Provincial People's Hospital Zhengzhou University Zhengzhou 450003 China; ^6^ Cancer Center Union Hospital Tongji Medical College Huazhong University of Science and Technology Wuhan 430022 China; ^7^ Institute of Radiation Oncology Union Hospital Tongji Medical College Huazhong University of Science and Technology Wuhan 430022 China; ^8^ Key Laboratory of Animal Models and Human Disease Mechanisms of Yunnan Province Kunming Institute of Zoology Chinese Academy of Sciences Kunming 650201 China

**Keywords:** basal like breast cancer, cell cycle, KLF5, RB1, XPO1

## Abstract

Basal‐like breast cancer (BLBC), overlapping with the subgroup of estrogen receptor (ER), progesterone receptor (PR), and HER2 triple‐negative breast cancer, has the worst prognosis and limited therapeutics. The *XPO1* gene encodes nuclear export protein 1, a promising anticancer target which mediates nucleus‐cytoplasm transport of nuclear export signal containing proteins such as tumor suppressor RB1 and some RNAs. Despite drugs targeting XPO1 are used in clinical, the regulation of *XPO1* expression and functional mechanism is poorly understood, especially in BLBC. This study finds that KLF5 is a transcription factor of *XPO1*, which increases RB1 nuclear export and cell proliferation in BLBC cells. Furthermore, XPO1 interacts with the RNA‐binding protein PTBP1 to export *FOXO1* mRNA to cytoplasm and thus activates the FOXO1‐KLF5 axis as a feedback. This work demonstrates that XPO1 inhibitor KPT‐330 in combination with CDK4/6 inhibitor additively suppressed BLBC tumor growth in vivo. These results reveal a novel positive feedback regulation loop between KLF5 and XPO1 and provide a novel treatment strategy for BLBC.

## Graphical abstract

KLF5 is highly expressed in BLBC patients and promotes Cyclin D1 and XPO1 transcription, ultimately leading to the inactivation of RB1. Both XPO1 inhibitor (KPT‐330) and CDK4/6 inhibitor (Palbociclib) can enhance RB1 activity and exert antitumor effects. It has been proved that the combination of KPT‐330 and Palbociclib can inhibit the proliferation of BLBC cells both in vitro and in vivo, and this medication strategy has high clinical application value.

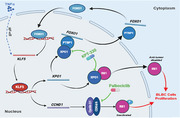

## 1 Introduction

Breast cancer (BC) is the most prevalent malignant tumor in women globally and one of the major causes of cancer‐related death in women.^[^ ^1^ ^]^ Breast cancer is divided into four subtypes based on IHC results for estrogen receptor (ERα), progesterone receptor (PR), human epidermal growth factor receptor‐2 (HER2), and the tumor cell proliferation index (Ki‐67):^[^ ^2^ ^]^ Luminal A, Luminal B, HER2 positive and basal‐like breast cancer (BLBC). The first‐line treatment of Luminal breast cancer is endocrine therapy combined with CDK4/6 inhibitor, which has a good response effect in patients.^[^ ^3^ ^]^ BLBC does not express ER, PR, or HER2. It accounts for ≈20% of all breast cancer cases and is more common in young women under the age of 40,^[^ ^4^ ^]^ and BLBC tends to be highly invasive, with a poor prognosis for patients.^[^ ^5^ ^]^ BLBC patients are most commonly treated with surgery, radiotherapy, and chemotherapy.^[^ ^6^ ^]^ PARP inhibitors have been approved to treat Homologous Recombination Deficiency (HRD) BLBC patients.^[^ ^7^ ^]^ Sacituzumab Govitecan(SG) targets the Trop‐2 protein of cancer cells to deliver irinotecan analogue SN‐38.^[^ ^8^ ^]^ These two drugs are suitable for a subset of BLBC, but the current BLBC is still mainly treated with radiotherapy and chemotherapy.^[^ ^9^ ^]^ It is important to develop novel therapeutics for BLBC patients.

The KLF5 transcription factor is highly expressed in BLBC.^[^ ^10^ ^]^ Our previous studies showed that KLF5 promotes breast cancer cell cycle progression and tumor growth through upregulating the transcription of *Cyclin D1*, *FGF‐BP1*, *mPGES1*, and *TNFAIP2* ^[^ ^11^, ^12^, ^13^ ^]^ and downregulating the transcription of cell cycle inhibitors, including *p21* and *p27*.^[^ ^13^, ^14^ ^]^ It is well known the Cyclin D1‐CDK4/6 complex phosphorylates RB1 and releases E2F transcription factors to drive the G1/S cell cycle progression.^[^ ^15^ ^]^ Consistently, KLF5 predominately promotes the G1/S cell cycle progression.^[^ ^16^, ^17^ ^]^ However, it is unclear why CDK4/6 inhibitors did not show good efficacy in BLBC.

The *XPO1* gene encodes nuclear export protein 1 (Exportin 1), or chromosomal region maintenance protein 1 (CRM1, XPO1). It belongs to the importin‐β superfamily of nuclear transport proteins. It can transport at least 221 proteins with nuclear export signal (NES) domains from the cell nucleus to the cytoplasm, such as RB1, p53, p21, Beclin1 and p27 tumor suppressors. When RB1 is transported out of the nucleus, it can no longer inhibit E2Fs and cell cycle progression.^[^ ^18^, ^19^ ^]^ Furthermore, XPO1 can transport certain RNA, including mRNA, miRNA, rRNA, *etc*. out of nucleus with the help of adaptor proteins.^[^ ^19^ ^]^ XPO1 is highly expressed in a variety of tumors, such as small cell lung cancer, glioma, cholangiocarcinoma, liver cancer, and so on.^[^ ^20^, ^21^, ^22^ ^]^ XPO1 inhibitor KPT‐330 (Selinexor) has been reported to inhibit multiple myeloma and diffuse large B‐cell lymphoma,^[^ ^23^ ^]^ colorectal cancer, and neuroblastoma.^[^ ^24^, ^25^ ^]^ Moreover, Selinexor has been approved for the clinical treatment of myeloma and diffuse large B‐cell lymphoma with good efficacy.^[^ ^26^ ^]^ It has been reported that XPO1 expression is increased in BLBC patients and KPT‐330 has anti‐tumor activity in BLBC cells.^[^ ^27^, ^28^ ^]^ The combination of KPT‐330 and PI3K/mTOR inhibitor GSK2126458 inhibited BLBC tumor growth.^[^ ^29^ ^]^ In a Phase Ib clinical trial with BLBC patients, the combination of Selinexor and Eribulin had a more significant response.^[^ ^30^ ^]^

In this study, we revealed a novel mechanism by which KLF5 promotes BLBC cell proliferation through inducing the *XPO1* gene transcription and XPO1 also increases the KLF5 expression through increasing the *FOXO1* mRNA nuclear export. Thus, a positive feedback regulation loop between KLF5 and XPO1 was established in BLBC. We further found that KLF5, on the one side, promotes *CCND1* (Encoding the Cyclin D1 protein) gene transcription and RB1 phosphorylation,^[^ ^13^ ^]^ on the other side, KLF5 promotes *XPO1* gene transcription and RB1 nuclear export. Both mechanisms work together to promote BLBC cell cycle progression. Consistently, a combination of XPO1 inhibitor KPT‐330 and CDK4/6 inhibitor Palbociclib effectively inhibited BLBC cell proliferation and tumor growth. Our results reveal that XPO1 may be a therapeutic target for BLBC and provide a novel combination therapy strategy for BLBC patients.

## 2 Results

### 2.1 XPO1 Promotes BLBC Cell Proliferation

**Figure 1:**
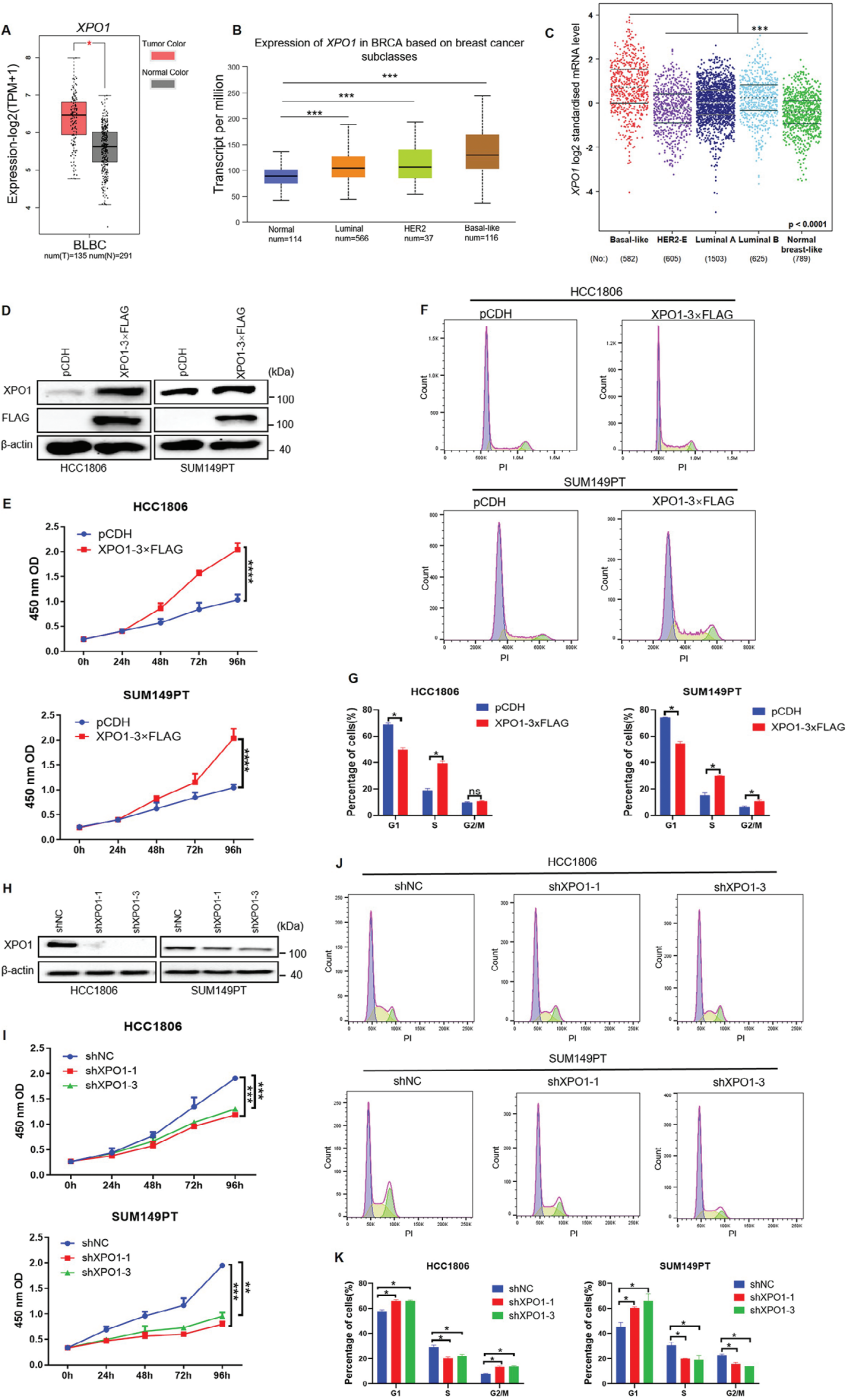
XPO1 promotes BLBC cell proliferation. A) The mRNA expression levels of *XPO1* in BLBC clinical samples from GEPIA online database. *p < 0.05.B) The mRNA expression levels of *XPO1* in different types of breast cancer from TCGA database. ***p < 0.001.C) The bc‐GenExMiner v4.5 online database was used to analyze *XPO1* mRNA expression levels in different breast cancer subtypes. ***p < 0.001. D) The XPO1 protein overexpression in BLBC cells was detected by WB. E) The activity of cells was detected by CCK‐8 assay after XPO1 overexpression(n = 3). ****p < 0.0001. F) XPO1 overexpression in BLBC cells accelerated the cell cycle progression, as detected by flow cytometry (n = 3). G) Quotative results of panel F. *p < 0.05, n. s, not significant. H) XPO1 stable knocked down by shRNA in BLBC cells was detected by WB. I) XPO1 knockdown in BLBC reduced cell viability, as detected by CCK‐8 assays (n = 3). **p <0.01, ***p < 0.001. J) XPO1 knockdown in BLBC cells blocked the cell cycle progression, as detected by flow cytometry (n = 3). K) Quotative results of panel J. *p < 0.05.

XPO1 is highly expressed in a variety of solid tumors, such as small cell lung cancer, cholangiocarcinoma and liver cancer, which is associated with a poor clinical outcome.^[^ ^19^ ^]^ By analyzing the expression of *XPO1* in BLBC through GEPIA and TCGA online databases, we found that compared with normal breast tissue, *XPO1* mRNA is also highly expressed in BLBC tumor tissues (**Figure** **1**A,B). Further analysis of the TCGA database showed that *XPO1* mRNA was particularly highly expressed in BLBC compared with other subtypes (Figure 1C). Our previous study showed that KLF5 was highly expressed in BLBC cell lines, including HCC1806, HCC1937 and SUM149PT.^[^ ^31^ ^]^ We found that *KLF5* and *XPO1* was also highly expressed in these three cell lines by the Human Protein Atlas database query (Figure S1A,B, Supporting Information), and by RT‐qPCR detection (Figure S1C, Supporting Information). The results showed that the expression levels of *KLF5* and *XPO1* in HCC1806 and SUM149PT cell lines were higher than that in HCC1937 cell line (Figure S1C, Supporting Information); therefore, HCC1806 and SUM149PT cell lines were selected for subsequent experiments. To evaluate the function of XPO1 in BLBC, we performed an RNA‐sequencing (RNA‐seq) analysis of XPO1 knockdown HCC1806 cells followed by a gene set enrichment analysis (GSEA), which revealed that XPO1 knockdown could inhibit cell cycle progression (Figures S1D, Supporting Information). Moreover, overexpression of XPO1 in HCC1806 and SUM149PT (Figure 1D) substantially promoted cell proliferation (Figure 1E) and colony formation (Figure S1E,F, Supporting Information). The flow cytometry results showed that XPO1 accelerated the transition from the G1 phase to the S phase (Figure 1F,G). In addition, XPO1 knockdown in BLBC cells resulted in the opposite phenotypes. Stable knockdown of XPO1 in BLBC cells by two different shRNA (Figure 1H and Figure S1G, Supporting Information), significantly suppressed cell growth (Figure 1I) and colony formation (Figure S1H,I, Supporting Information), decreased the number of cells in the S phase and the number of cells in G1 phase increased (Figure 1J,K), leading to cell proliferation inhibition. These results suggest that XPO1 is highly expressed in BLBC and promotes BLBC G1/S cell cycle progression.

### 2.2 KLF5 Promotes *XPO1* Gene Transcription and Cell Proliferation through XPO1 in BLBC

**Figure 2:**
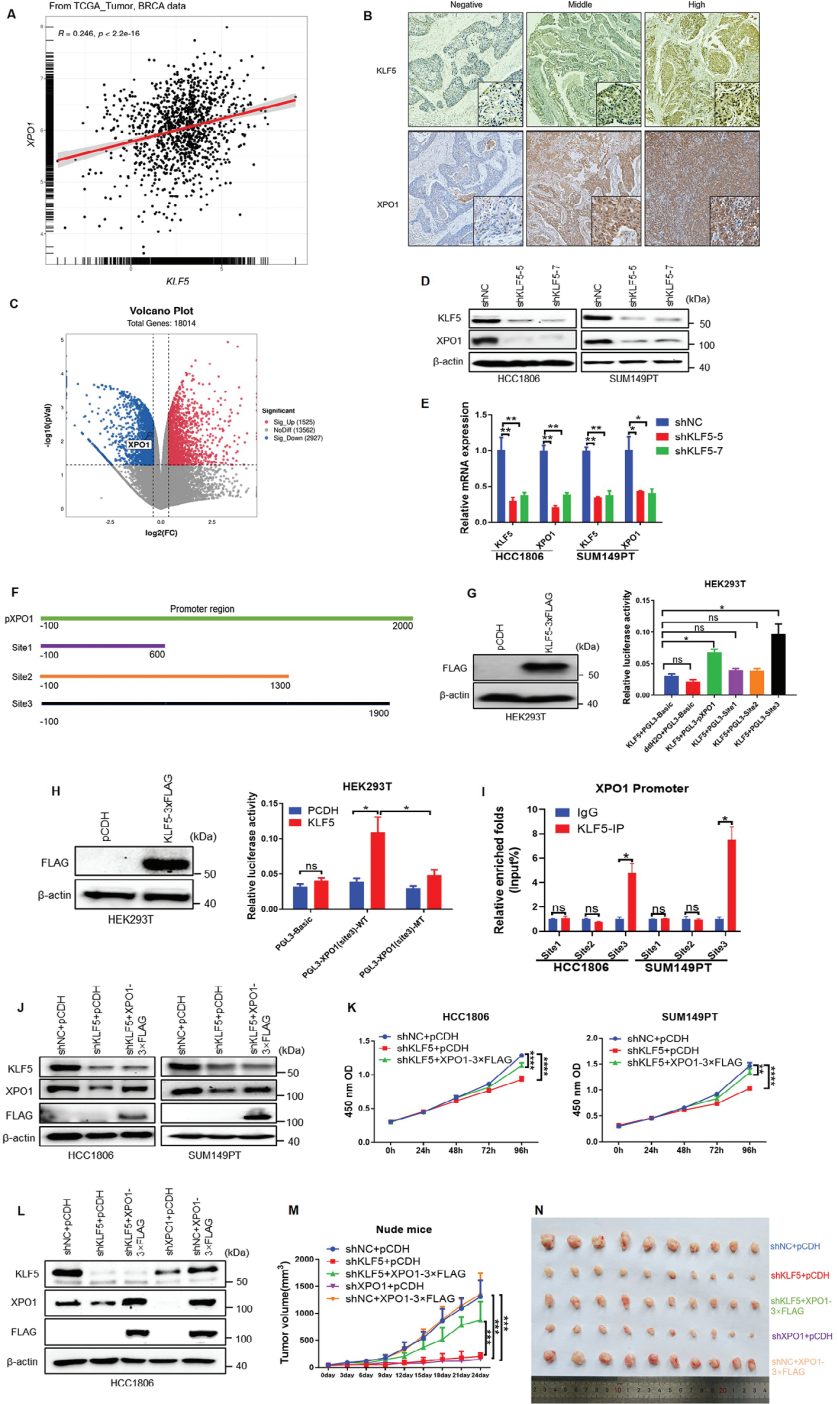
KLF5 promotes *XPO1* gene transcription and cell proliferation through XPO1 in BLBC. A) The positive association between *KLF5* and *XPO1* mRNA expression levels in clinical BLBC samples from the TCGA database. B) The expression of KLF5 and XPO1 proteins in BLBC patient tissue samples, as assessed by immunohistochemical (IHC) staining. The images are shown at 5× and 40× magnification (n = 87). C) RNA‐seq data suggest that XPO1 mRNA was positively regulated by KLF5 in HCC1806 cells. D) KLF5 knockdown decreased the XPO1 protein levels in BLBC cells. The protein expression levels of XPO1 were detected by WB. E) KLF5 knockdown decreased the *XPO1* mRNA levels in BLBC cells. The mRNA expression levels of *XPO1* were detected by RT‐qPCR (n = 3). *p < 0.05, **p < 0.01. F) Dual luciferase reporter plasmids of the full‐length and truncated *XPO1* gene promoter were constructed, respectively, and named as, pXPO1, Site1, Site2, Site3. G) The binding of KLF5 to site3 at the *XPO1* gene promoter was detected by dual luciferase reporter assays (n = 3). *p < 0.05, n. s, not significant. H) The site3 mutation abolished the activation of the *XPO1* gene promoter by KLF5, as detected by dual luciferase reporter assays (n = 3). *p < 0.05, n. s, not significant. I) The binding sites of KLF5 and XPO1 promoter region were detected by ChIP‐qPCR (n = 3). *p < 0.05, n. s, not significant. J) KLF5 knockdown and XPO1 overexpression in BLBC cells were detected by WB. K) XPO1 overexpression can partially rescue KLF5 knockdown induced cell growth inhibition. CCK‐8 assay was used to detect the cell viability (n = 3). **p < 0.01, ****p < 0.0001. L) WB was used to verify the effect of KLF5 knockdown and XPO1 overexpression in HCC1806 cells. M) XPO1 overexpression can partially rescue KLF5 knockdown induced tumor growth inhibition, as measured by tumor volume changes (n = 10). ***p < 0.001. N) XPO1 overexpression can partially rescue KLF5 knockdown induced tumor growth inhibition. Tumor masses were obtained from nude mice.

The GEPIA database revealed that *KLF5* was predominately expressed in BLBC (Figure S2A, Supporting Information). The TCGA database also revealed that *KLF5* and *XPO1* were highly expressed in BLBC (Figure S2B,C, Supporting Information). Importantly, there was a significant correlation between the mRNA expression levels of *KLF5* and *XPO1* in BLBC cell lines (Figure S2D, Supporting Information). Consistently, the mRNA expression levels of *XPO1* and *KLF5* are positively correlated in breast cancer clinical samples (**Figure** **2**A), particularly in BLBC patients (Figure S2E–J, Supporting Information). We performed IHC in 87 BLBC clinical samples (Figure 2B) and validated a positive correlation between KLF5 and XPO1 protein expression (*R* = 0.3179, *p <* 0.0001), with a 77% double‐positive rate (Figure S2K, Supporting Information). RNA‐seq analysis of KLF5 knockdown HCC1806 cells revealed that *XPO1* mRNA expression was reduced (Figure 2C). Indeed, KLF5 knocked down in HCC1806 and SUM149PT cells resulted in decreased *XPO1* mRNA and protein expression levels (Figure 2D,E and Figure S2L,M, Supporting Information). To test whether KLF5 directly promotes *XPO1* gene transcription, we analyzed the *XPO1* gene promoter using the JASPAR online tool and identified three putative KLF5 binding sites (Figures S2N, Supporting Information), Then the reporter plasmids of the full length of the *XPO1* gene promoter region and three prediction sites were constructed (Figure 2F). Luciferase reporter assays revealed that KLF5 activates the *XPO1* gene promoter through site 3 (Figure 2G). Next, we mutated site 3 (Figure S2O, Supporting Information), and found that the mutation abolished KLF5‐induced promoter activation (Figure 2H). These results revealed that KLF5 binds to site 3 of the *XPO1* gene promoter to promote the *XPO1* transcription. In addition, ChIP assay revealed that KLF5 indeed binds to XPO1 site 3, not site 1 and site 2 (Figure 2I and Figure S2P, Supporting Information).

To test whether KLF5 promotes cell proliferation through XPO1, we performed a rescue experiment in which KLF5 was knocked down, and then XPO1 was overexpressed in HCC1806 and SUM149PT cells (Figure 2J). As expected, XPO1 overexpression partially restored cell proliferation caused by KLF5 knockdown (Figure 2K). Consistently, in vivo animal experimental results also showed that KLF5 promotes tumor growth through *XPO1*, at least in part (Figure 2L–N).

### 2.3 XPO1 Promotes BLBC Cell Proliferation by Transporting RB1 Protein out of the Nucleus

**Figure 3:**
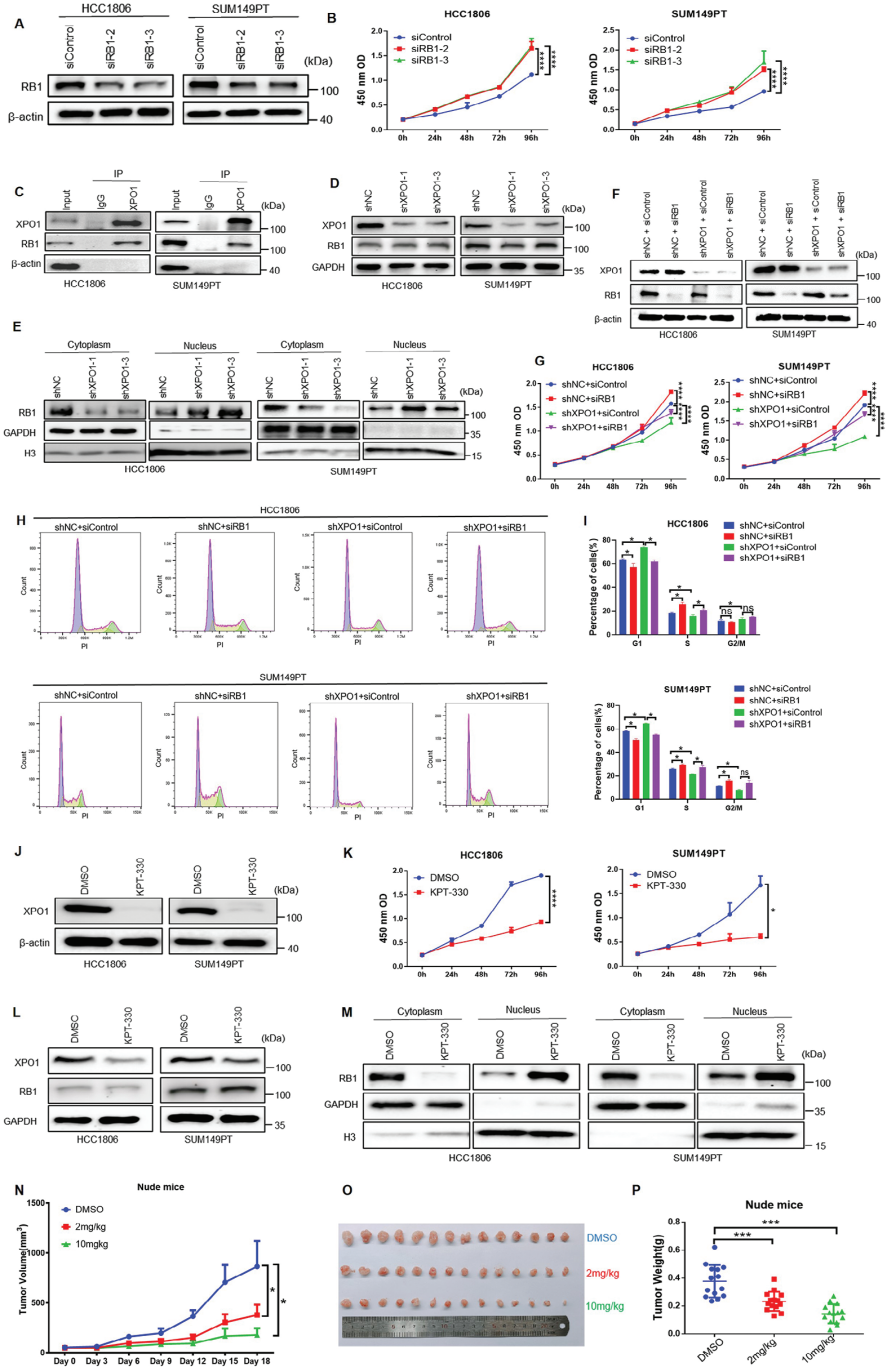
XPO1 promotes BLBC cell proliferation by transporting RB1 protein out of the nucleus. A) RB1 knockdown in BLBC cells was detected by WB. B) RB1 knockdown promoted BLBC cell proliferation, as measured by the CCK‐8 assays (n = 3). ***p < 0.001, ****p < 0.0001. C) Endogenous XPO1 protein interacts with RB1protein, as measured by Co‐IP assays in BLBC cells. D) XPO1 knockdown did not affect the expression levels of total RB1 protein in BLBC cells, as detected by WB. E) XPO1 knockdown increased the nuclear localization of RB1. Nuclear and cytoplasmic fractionations were collected for WB. F) Knockdown of XPO1 and RB1 in BLBC cells were detected by WB. G) Knockdown of RB1 rescued the cell growth inhibition induced by XPO1 knockdown in BLBC cells, as assessed by CCK‐8 cell growth assays (n = 3). ****p < 0.0001. H) Knockdown of RB1 rescued the cell cycle inhibition induced by XPO1 knockdown in BLBC cells. Cell cycle changes were detected by flow cytometry (n = 3). I) Quotative results of panel H. *p < 0.05, n. s, not significant. J) XPO1 inhibitor (KPT‐330) decreased the XPO1 protein expression levels in BLBC cells, as detected by WB. K) KPT‐330 inhibited BLBC cell growth, as assessed by CCK‐8 cell growth assays (n = 3). *p < 0.05, ****p < 0.0001. L) KPT‐330 did not affect the expression levels of total RB1 protein in BLBC cells, as detected by WB. M) KPT‐330 increased the nuclear accumulation of RB1. N) KPT‐330 significantly inhibited the growth of tumor cells. The tumor volume of nude mice was measured once every 3 days (n = 7). *p < 0.05. O) Treatment of BLBC with KPT‐330 for 18 days resulted in a significant reduction in tumor mass (n = 14). P) Quotative results of panel O. ***p < 0.001.

RB1 exerts its tumor suppressor function by binding to E2Fs in the nucleus. Nuclear export and phosphorylation are two major approaches causing the loss of RB1's tumor suppressor function.^[^ ^25^, ^32^ ^]^ XPO1 was reported to promote the proliferation of glioma cells by increasing the nuclear export of RB1.^[^ ^25^ ^]^ To validated whether XPO1 functions through RB1 nuclear export in BLBC cells, we knocked down wild‐type RB1^[^ ^33^ ^]^ in HCC1806 and SUM149PT cells and found that cell proliferation was significantly increased (**Figure** **3**A,B). Then, by immunofluorescence assay, we found that XPO1 and RB1 proteins were both expressed in the nucleus and cytoplasm, both are mainly expressed in the nucleus. (Figure S3A, Supporting Information). Next, we confirmed that exogenous (Figure S3B, Supporting Information) and endogenous (Figures 3C) XPO1 interacts with RB1 in BLBC cells. XPO1 knockdown in HCC1806 and SUM149PT cells did not influence total RB1 protein expression (Figure 3D). Notably, XPO1 knockdown resulted in an obvious reduction in RB1 expression in the cytoplasm and an increased RB1 expression in the nucleus (Figure 3E), we selected GAPDH protein as a cytosolic marker and H3 protein as a nuclear marker. These results showed that XPO1 can increase the nuclear export of RB1 in BLBC cells. To test whether XPO1 promotes cell proliferation through RB1 in BLBC, we knocked down XPO1 and RB1 in HCC1806 and SUM149PT cells (Figure 3F) and found that knockdown of RB1 could partly restore the cell proliferation induced by XPO1 knockdown (Figure 3G). We performed a flow cytometry assay and found that knockdown of RB1 could significantly rescue the G1 phase cell increase and S phase cell decrease caused by XPO1 knockdown (Figure 3H,I).

KPT‐330 is an XPO1 inhibitor, it can compete with proteins which containing NES domain to bind XPO1, so that XPO1 cannot transport cargo proteins out of the nucleus, at the same time, when XPO1 binds to KPT‐330, KPT‐330 can promote its degradation.^[^ ^22^ ^]^ KPT‐330 used in clinical practice to treat multiple myeloma and has good anti‐tumor activity.^[^ ^18^ ^]^ It was shown that KPT‐330, alone or in combination, had a therapeutic impact in patients with advanced BLBC, but the efficacy is limited.^[^ ^30^, ^34^ ^]^ In HCC1806 and SUM149PT cells, KPT‐330 inhibited the XPO1 expression and cell proliferation (Figure 3J,K and Figure S3C,D, Supporting Information). The flow cytometry results showed that KPT‐330 induced cell cycle arrest in the G1 phase (Figure S3E,F, Supporting Information), which is consistent with the results of XPO1 knockdown (Figure 1J,K). Consistently, KPT‐330 did not change the total RB1 protein expression level, but the nuclear RB1 protein levels were increased (Figure 3L,M). In addition, KPT‐330 showed strong anti‐tumor activity in vivo (Figure 3N–P). KPT‐330 did not significantly influence animal body weight during administration or liver function in nude mice (Figure S3G–I, Supporting Information), and there was no obvious damage to the organs of nude mice (Figure S3J, Supporting Information). We also performed IHC staining of RB1 in BLBC samples from clinical samples, and correlation analysis of cytoplasmic localization of total XPO1 and RB1 was performed. It was found that the expression of XPO1 was positively correlated with the cytoplasmic expression of RB1 (*R* = 0.4620, *p <* 0.0001), with a 80.5% double‐positive rate (Figure S3K,L, Supporting Information). We have shown that KLF5 is a transcription factor for XPO1 (Figure 2), we performed a correlation analysis of the cytoplasmic localization of total KLF5 and RB1, and the IHC results showed that high KLF5 expression was associated with increased cytoplasmic localization of RB1 (*R* = 0.2799, *P* = 0.0083), with a 69% double‐positive rate (Figure S3K,M, Supporting Information).

### 2.4 KLF5 Increases Cyclin D1 and XPO1 Expression to Promote RB1 Phosphorylation and Nuclear Export

**Figure 4:**
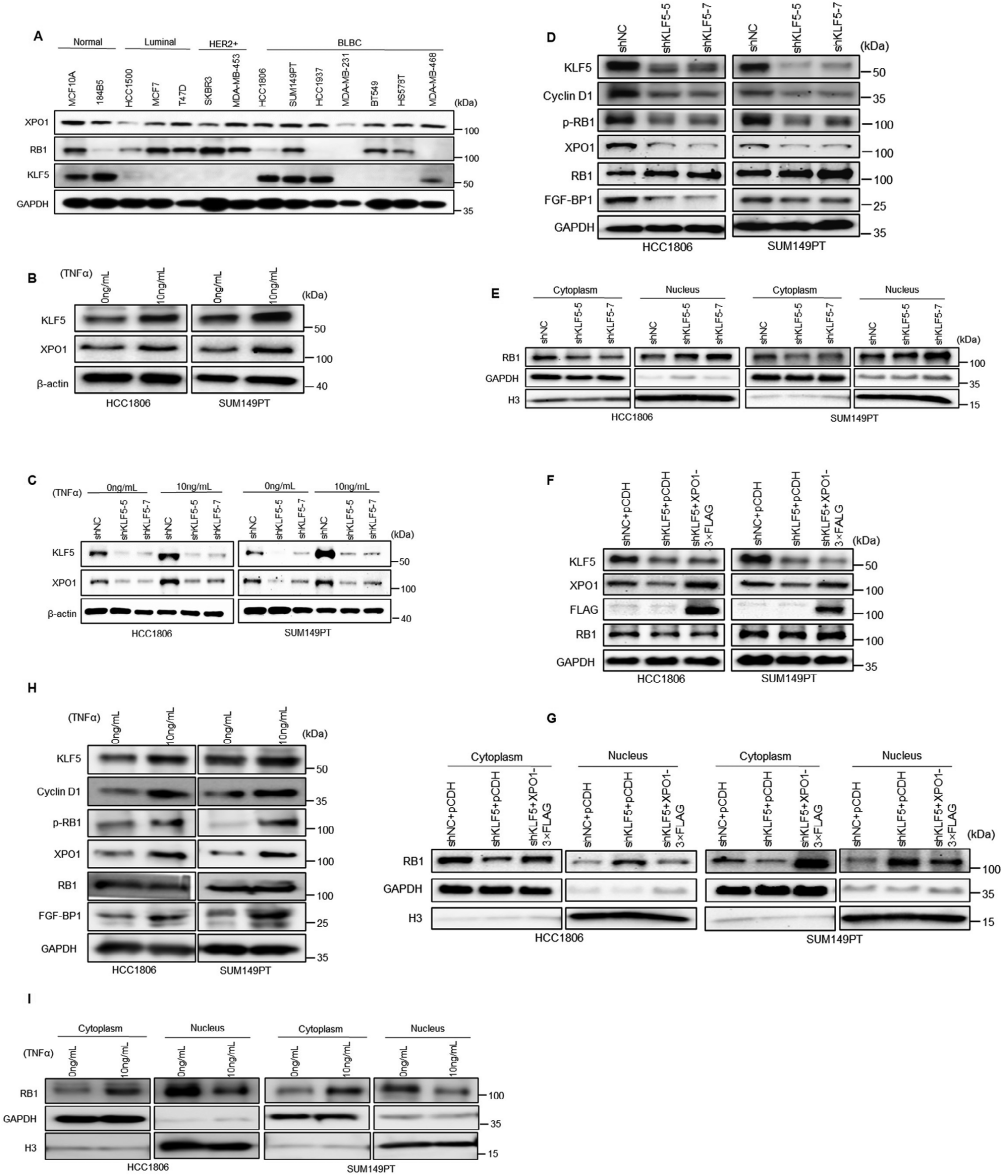
KLF5 increases Cyclin D1 and XPO1 expression to promote RB1 phosphorylation and nuclear export. A) The protein expression levels of XPO1, RB1 and KLF5 in normal breast epithelium and breast cancer cell lines, as measured by WB. B) TNFα induced both KLF5 and XPO1 protein expression in BLBC cells, as detected by WB. C) KLF5 is essential for TNFα to induce XPO1 protein expression in BLBC cells, as detected by WB. D) KLF5 knockdown decreased the protein expression levels of XPO1, Cyclin D1, FGF‐BP1 and p‐RB1 in BLBC cell lines, as detected by WB. E) KLF5 knockdown increased the nuclear localization of RB1, as detected by WB. F) KLF5 knockdown and XPO1 overexpression in BLBC cells were verified by WB. G) XPO1 overexpression rescued KLF5 knockdown induced the nuclear localization of RB1, as detected by WB. H) TNFα increased the protein expression of Cyclin D1, pRB1, XPO1 and FGF‐BP1 in BLBC cells, as detected by WB. I) After TNFα stimulation of BLBC cells, nuclear export of RB1 was promoted, Nuclear and cytoplasmic fractionations were collected for WB.

RB1 activity is frequently lost in breast cancer because of phosphorylation by Cyclin D1‐CDK4/6 complex.^[^ ^13^, ^35^ ^]^ Our previous study suggested that KLF5 can increase *Cyclin D1* transcription.^[^ ^13^, ^36^, ^37^ ^]^ KLF5, XPO1, and RB1 were all expressed in the HCC1806 and SUM149PT cell lines (**Figure** **4**A). Our previous study showed that TNFα can stimulate KLF5 expression.^[^ ^38^, ^39^ ^]^ Consistently, TNFα increased the XPO1 protein expression in HCC1806 and SUM149PT cells (Figure 4B). When KLF5 was silenced, TNFα could no longer induce XPO1 protein expression (Figure 4C); therefore, TNFα promotes XPO1 expression through KLF5. To test whether KLF5 increase RB1's phosphorylation, we knocked down KLF5 in BLBC cells and found that the Cyclin D1 and p‐RB1protein levels were decreased, and the total RB1 protein level did not significantly alter (Figure 4D). Since KLF5 is a transcription factor for *XPO1* (Figure 2) and that XPO1 can transport RB1 protein out of the nucleus (Figure 3), we wondered whether KLF5 promotes RB1 cytoplasmic localization via XPO1. Notably, KLF5 knockdown increased RB1 nuclear accumulation in HCC1806 and SUM149PT cells (Figure 4E). Additionally, XPO1 overexpression in KLF5‐knockdown BLBC cells (Figure 4F) resulted in partial recovery nuclear export of RB1 (Figure 4G). In addition, treatment of BLBC cells with TNFα increased the protein level of Cyclin D1 and the phosphorylation level of RB1 (Figure 4H). The nuclear export of RB1 was also enhanced by TNFα treatment (Figure 4I). The most important role of RB1 is to inhibit cycle progression by inhibiting E2F release, so does KLF5 affect cycle directly by regulating E2F1? KLF5 knockdown in BLBC, and the WB results showed that E2F1 did not change significantly (Figure S3N, Supporting Information). Combined with the previous results, we can conclude that TNFα can increase the expression of KLF5, and its downstream target genes Cyclin D1 and XPO1 may be up‐regulated after the increase of KLF5 expression, increase RB1 both phosphorylation and nuclear export, leading to RB1 inactivation and cell proliferation.

### 2.5 XPO1 Promotes KLF5 Expression by Binding to PTBP1 and *FOXO1* mRNA Nuclear Export in BLBC Cells

**Figure 5:**
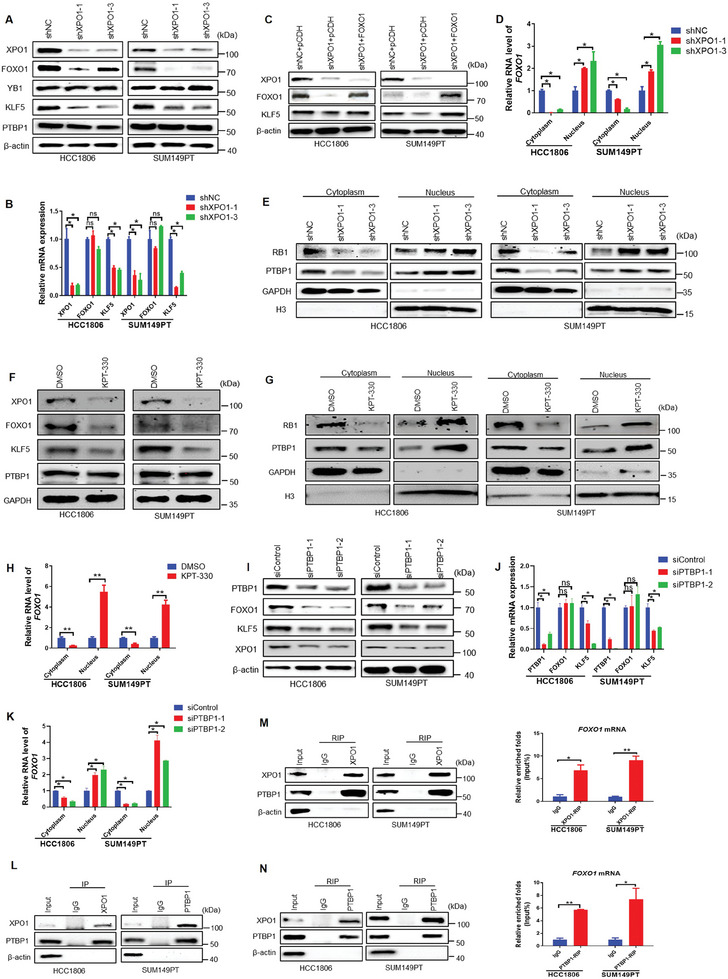
XPO1 promotes KLF5 expression by binding to PTBP1 and *FOXO1* mRNA nuclear export in BLBC cells. A) XPO1 knockdown decreased the protein expression levels of FOXO1 and KLF5, but did not affect the protein expression levels of YB1 and PTBP1 in BLBC cells, as detected by WB. B) XPO1 knockdown decreased the mRNA expression levels of *KLF5*, but did not affect the mRNA expression levels of *FOXO1* in BLBC cells, as detected by RT‐qPCR (n = 3). *p < 0.05, n. s, not significant. C) FOXO1 overexpression rescued XPO1 knockdown induced KLF5 expression decrease in BLBC cells, as detected by WB. D) XPO1 knockdown in BLBC cells increased the nuclear localization of *FOXO1* mRNA, as detected by RT‐qPCR (n = 3). *p < 0.05. E) XPO1 knockdown in BLBC cells increased the nuclear localization of PTBP1 protein, as detected by WB. F) KPT‐330 decreased the total protein expression levels of FOXO1 and KLF5 in BLBC cells, as detected by WB. G) KPT‐330 increased the nuclear localization of PTBP1 and RB1 proteins, as detected by WB. H) KPT‐330 increased the nuclear localization of *FOXO1* mRNA, as determined by RT‐qPCR (n = 3). **p < 0.01. I) PTBP1 knockdown decreased the protein expression levels of FOXO1, KLF5 and XPO1, as detected by WB. J) PTBP1 knockdown decreased the mRNA expression levels of *KLF5*, but not *FOXO1*, as detected by RT‐qPCR (n = 3). *p < 0.05, n. s, not significant. K) PTBP1 knockdown increased the nuclear localization of *FOXO1* mRNA, as determined by RT‐qPCR (n = 3). *p < 0.05. L) The binding between the endogenous XPO1 and PTBP1 proteins was examined by Co‐IP assays in BLBC cells. M) XPO1 bound to the *FOXO1* mRNA and PTBP1 protein in BLBC cells. IP was performed with the anti‐XPO1 Ab. Proteins were examined by WB and mRNA was detected by RT‐qPCR (n = 3). *p < 0.05, **p < 0.01. N) PTBP1 bound to the *FOXO1* mRNA and XPO1 protein in BLBC cells. IP was performed with the anti‐PTBP1 Ab. Proteins were examined by WB and mRNA was detected by RT‐qPCR (n = 3). *p < 0.05, **p < 0.01.

Since KLF5 promotes XPO1 expression, we wondered whether XPO1 also regulate KLF5 expression. We knocked down XPO1 in HCC1806 and SUM149PT cells and found that the KLF5 protein and mRNA expression levels were decreased (**Figure** **5**A,B), meanwhile, the expression of the downstream target genes of KLF5 was also decreased (Figure S4A, Supporting Information). Our previous studies showed that KLF5 was positively regulated by FOXO1 and YB1 transcription factors, FOXO1 is a direct transcription factor for KLF5, and YB1 is an mRNA stabilizing factor for KLF5.^[^ ^31^, ^40^ ^]^ XPO1 knockdown resulted in a significant decrease of FOXO1, but not YB1, protein levels in BLBC cells (Figure 5A). Interestingly, *FOXO1* mRNA levels were not changed significantly by XPO1 knockdown (Figure 5B). Importantly, FOXO1 overexpression could restore the KLF5 expression decrease induced by XPO1 knockdown (Figure 5C), suggesting that XPO1 positively regulates the *KLF5* transcription through FOXO1.

It has been reported that XPO1 can interact with certain RNA‐binding proteins to co‐transport certain oncogenic mRNAs out of the nucleus to promote their translation.^[^ ^41^ ^]^ It has been reported that XPO1 can bind to the RNA‐binding protein PTBP1,^[^ ^42^ ^]^ which can bind to *FOXO1* mRNA.^[^^]^ We hypothesized that XPO1 promotes the translocation of *FOXO1* mRNA and increase KLF5 expression by transporting *FOXO1* mRNA out of the nucleus with the help of PTBP1. As expected, knockdown of XPO1 in HCC1806 and SUM149PT cells resulted in increased nuclear localization of PTBP1 protein and *FOXO1* mRNA (Figure 5D,E). KPT‐330 could also suppress the expression of FOXO1 and KLF5 by increasing the nucleus accumulation of PTBP1 protein and *FOXO1* mRNA (Figure 5F–H). In agreement with our hypothesis, PTBP1 knockdown also resulted in decreased protein expression of FOXO1, KLF5 and XPO1 in both BLBC cell lines (Figure 5I). In addition, PTBP1 knockdown did not affect total *FOXO1* mRNA expression, but decreased *KLF5* mRNA expression and increased *FOXO1* mRNA nuclear accumulation (Figures 5J‐K). XPO1 knockdown did not affect total PTBP1 expression levels (Figure 5A). Then, by immunofluorescence assay, we found that XPO1 and PTBP1 were both expressed in the nucleus and cytoplasm, both are mainly expressed in the nucleus. (Figure S4B, Supporting Information). Next, we confirmed that endogenous XPO1 and FOXO1 proteins interacted with each other in BLBC cells (Figure 5L). These results suggest that XPO1 binds to PTBP1 to promote KLF5 transcriptional expression via increasing the nuclear export of *FOXO1* mRNA.

Next, we tested whether XPO1 and PTBP1 bind to *FOXO1* mRNA. Indeed, *FOXO1* mRNA were co‐immunoprecipitated with XPO1 or PTBP1 proteins by RNA immunoprecipitation (RIP) assays (Figure 5M,N). Then, we performed RIP‐PCR experiments again using XPO1 and PTBP1 antibodies separately, the conclusion was same with the RIP assay (Figure S4C–E, Supporting Information). Finally, we overexpressed XPO1‐3xFLAG followed by knockdown of PTBP1, and the FLAG‐RIP results showed that the FOXO1 mRNA enrichment rate was decreased after PTBP1 knockdown (Figure S4F, Supporting Information). These results reveal that XPO1 interacts with RNA‐binding protein PTBP1 in BLBC cells to promote the export of *FOXO1* mRNA from the nucleus, enhance the translation of FOXO1 protein, and consequently increase KLF5 expression.

### 2.6 CDK4/6 Inhibitor Palbociclib, in Combination with XPO1 Inhibitor KPT‐330, Shows an Additive Therapeutic Effect on BLBC

**Figure 6:**
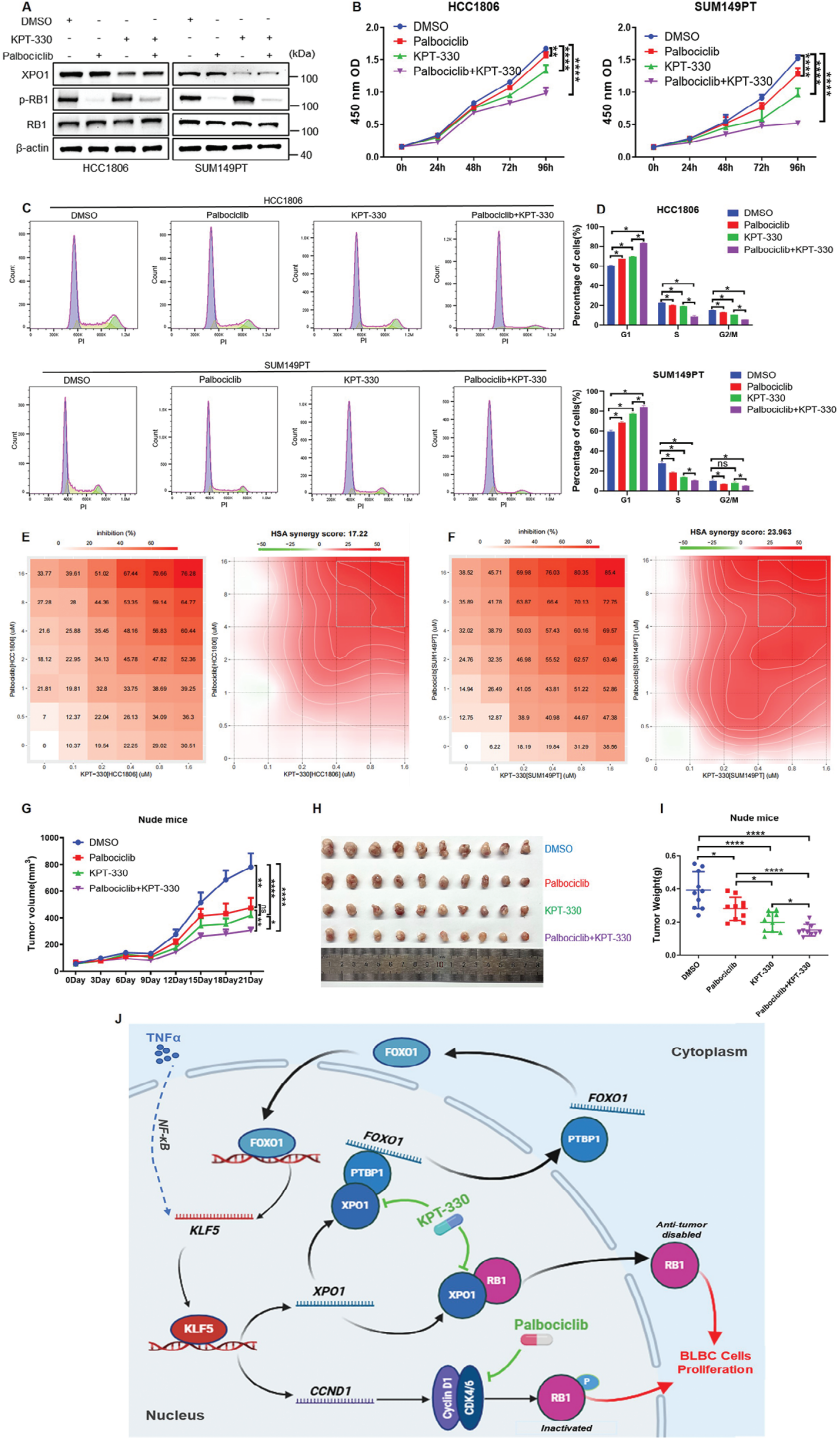
CDK4/6 inhibitor Palbociclib, in combination with XPO1 inhibitor KPT‐330, has an additive therapeutic effect on BLBC. A) The expression levels of XPO1, p‐RB1 and RB1 proteins in BLBC cells treated with CDK4/6 inhibitor (Palbociclib) and KPT‐330 were detected by WB. B) The combination of Palbociclib and KPT‐330 additively inhibited BLBC cell proliferation, as measured by CCK‐8 assays (n = 3). **p < 0.01, ****p < 0.0001. C) The combination of Palbociclib and KPT‐330 additively induced cell cycle G1 phase arrest. The cell cycle changes were detected by flow cytometry. D) Quotative results of panel C (n = 3). *p < 0.05, n. s, not significant. E, F) BLBC cells were treated with various concentrations of Palbociclib and KPT‐330 for 72h. The inhibition percentages of cell viabilities were measured by CCK‐8 assay (n = 3), and combination effects were analyzed using the Online Synergy Finder web application 3.0. G) The combination of Palbociclib and KPT‐330 can significantly inhibit the growth of BLBC tumors. BLBC nude mice were treated by combined administration for 21 days, and tumor volume was measured once every 3 days (n = 5). *p < 0.05, **p < 0.01, ****p < 0.001, n. s, not significant. H) Palbociclib combined with KPT‐330 can significantly reduce the tumor weight of BLBC in nude mice. After 21 days of combined treatment, the nude mice were sacrificed, and the tumor weight was taken (n = 10). I) Quotative results of panel F. *p < 0.05, ****p < 0.0001. J) Diagram of the regulatory mechanism between KLF5 and XPO1 in BLBC.

The Cyclin D1‐CDK4/6 complex phosphorylates RB1 and CDK4/6 inhibitors have been used to treat ER+ HER2‐ breast cancer, but not BLBC.^[^ ^3^ ^]^ Because the CDK4/6 inhibitor and the XPO1 inhibitor target a common protein RB1 and they have shown positive therapeutic effects in liver cancer and leukemia,^[^ ^44^, ^45^ ^]^ we predict that the combination of the two drugs will be effective in RB1 positive BLBC. We treated HCC1806 and SUM149PT cells with CDK4/6 inhibitors (Palbociclib, Ribociclib and Abemaciclib) and KPT‐330, they all showed good anti‐tumor activity, Abemaciclib was more effective than the other two drugs (Figure S5A, Supporting Information). Because Abemaciclib targets CDK4/6/9, Ribociclib shows similar antitumor efficacy as Palbociclib, and Palbociclib is less expensive and more commonly available, we selected palbociclib for the follow‐up study. Palbociclib and KPT‐330 treat BLBC cell lines (**Figure** **6**A) and result shows that the combination of the two drugs additively inhibited cell proliferation (Figure 6B), and flow cytometry results found that the combination of the two drugs could increase the number of cells in the G1 phase (Figures 6C,D). The synergistic effect of the two drugs was analyzed by synergy index, and the results showed that there was a synergistic effect between the two drugs (Figure 6E,F). The combination also showed an additive therapeutic effect on HCC1806 xenograft tumors in nude mice (Figure 6G–I and Figure S5B, Supporting Information). The combination of Palbociclib and KPT‐330 showed a low toxicity on animals based on body weight changes, liver function tests and morphology of organ tissues in nude mice (Figure S5C–E, Supporting Information).

## 3 Discussion

In this study, we revealed that KLF5 inhibited the RB1 activity and promoted cell proliferation in BLBC through two independent mechanisms. On the one side, KLF5 induces *Cyclin D1* gene transcriptional expression to phosphorylate RB1. On the other side, KLF5 induces *XPO1* gene transcriptional expression to export RB1 out of nucleus. Interestingly, XPO1 also induces the *KLF5* gene transcription by FOXO1 as a positive feedback loop. XPO1 exports *FOXO1* mRNA out of nucleus through interacting with PTBP1 protein. Most importantly, the combination of two clinical drugs (CDK4/6 inhibitor Palbociclib and XPO1 inhibitor KPT‐330) effectively inhibited BLBC cell proliferation and tumor growth (Figure 6H). These novel findings suggest that the combination of CDK4/6 inhibitor and XPO1 inhibitor may be a novel therapy strategy for BLBC patients.

Our previous studies have shown that KLF5 is an oncogenic transcription factor in BLBC^[^ ^31^, ^38^, ^46^, ^47^ ^]^ in response to pro‐inflammatory factor stimulation.^[^ ^31^ ^]^ However, the mechanism of action of KLF5 and the reason for its elevated expression in BLBC are not completely clear. In this study, we revealed that TNFα increased the expression of KLF5 and then induced XPO1 and Cyclin D1 expression, which promotes RB1 phosphorylation and nuclear export. Interestingly, XPO1 increased FOXO1‐mediated *KLF5* transcription. We previously reported that KLF5 induces *KPRT4* lncRNA transcription to promote YB1 mediated KLF5 transcription.^[^ ^48^ ^]^ These mechanisms ultimately maintain a high expression level of KLF5 and accelerate the proliferation of BLBC cells.

XPO1 is an oncoprotein in hematological tumor^[^ ^48^ ^]^ and a variety of solid tumors.^[^ ^19^ ^]^ Our results showed that XPO1 is highly expressed in BLBC, promotes the G1/S cell cycle progression and cell proliferation (Figure 1). We demonstrated that KLF5 is a transcription factor for XPO1 in BLBC, and KLF5 promotes cell proliferation by XPO1 (Figure 2). In agreement with this, the expression of KLF5 and XPO1 is positively correlated in BLBC clinical samples (Figure 2). XPO1 contains a particular NES binding domain, which can transport a number of proteins containing NES domains, including several tumor suppressors, such as RB1, p53, p21, and p27 out of the nucleus.^[^ ^22^, ^25^ ^]^ Thus, XPO1 inhibits their tumor suppressor activity by reducing their nuclear localization. Indeed, we found that XPO1 can exert a tumor‐promoting function by reducing the nuclear localization of RB1 (Figure 3). An XPO1 small molecule inhibitor (Selinexor or KPT‐330) has been shown to inhibit hematological tumor.^[^ ^49^, ^50^ ^]^ We confirmed that KPT‐330 has a significant inhibitory effect on the proliferation of BLBC in vitro and in vivo (Figure 3). It has been reported that the combination of KPT‐330 and mTOR inhibitor (GSK2126458) can inhibit the proliferation of BLBC cells.^[^ ^29^ ^]^ Recent clinical trial results showed that the combination of KPT‐330 and Eribulin has a better response effect in BLBC.^[^ ^30^ ^]^ Taken together, KPT‐330 may be used to treat BLBC alone or in combination.

RB1 tumor suppressor is commonly phosphorylated and deleted in BLBC.^[^ ^51^ ^]^ Palbociclib is a CDK4/6 inhibitor that inhibits RB1 phosphorylation. It is commonly used to treat ER+ and HER‐ breast cancer, but its efficacy in BLBC is not ideal.^[^ ^52^, ^53^ ^]^ It has been reported that knockdown of ACAA1 can increase the anti‐tumor activity of CDK4/6 inhibitors in BLBC.^[^ ^53^ ^]^ Mechanistically, the synergistic effect of ACAA1 knockdown and CDK4/6 inhibitors reduces the phosphorylation of RB1 and increases the tumor suppressor function of RB1.^[^ ^53^ ^]^ Our results reveal that KLF5 can promote BLBC cell proliferation by increasing the expression of both Cyclin D1 and XPO1, which increases RB1 phosphorylation and reduces RB1 nuclear localization (Figure 4). As RB1 is known to function by inhibiting E2F1 release, does KLF5 promote E2F1 expression at the transcriptional level? We found that KLF5 did not affect E2F1 expression, and therefore, we suggest that KLF5 may play a part in promoting cancer by inactivating RB1 by regulating the expression of Cyclin D 1 and XPO1 (Figure 4). In theory, KLF5 inhibitors should have a positive therapeutic effect on BLBC, but there is no KLF5 inhibitors in clinical practice. Therefore, we chose to use Palbociclib and KPT‐330 in combination to assess the therapeutic efficacy in BLBC. The combination additively inhibited BLBC cell proliferation and tumor growth (Figures 4 and 6). KPT‐330 was initially used in the treatment of leukemia with good efficacy.^[^ ^54^ ^]^ At present, the combination of KPT‐330 and other drugs has been tested in some clinical trials, such as: Ixazomib (a proteasome inhibitor) in advanced sarcoma (NCT03880123), paclitaxel and carboplatin in advanced ovarian and endometrial cancers (NCT02269293), gemcitabine and nab‐paclitaxel in pancreatic cancer (NCT02178436), and several standard chemotherapy regimens for treatment of various advanced solid tumors (NCT02419495).^[^ ^55^ ^]^ Our results may provide a theoretical basis for the investigator‐initiated test (IIT) clinical trial of KPT‐330 combined with Palbociclib in the treatment of BLBC. However, there are certain requirements for inclusion in clinical patients, for example, tumor tissue samples should have increased protein expression of KLF5, XPO1 and Cyclin D1, and no mutation of RB1 and positive expression in the cytoplasm. Although both KPT‐330 and Palbociclib have been used in clinical practice, no reports of the combination of the two drugs in the treatment of solid tumors have been reported. Although this study suggests that this treatment regimen can be applied to the treatment of patients with BLBC, studies on the optimal dose, associated toxicity, and long‐term tumor resistance of both drugs are lacking. Therefore, before clinical research, some basic research should be added to find the appropriate combination drug dose.

In addition to transporting tumor suppressor proteins out of the nucleus, XPO1 can interact with specific RNA‐binding proteins to co‐transport oncogene mRNA out of the nucleus, promote translation, and exert tumor‐promoting activity.^[^ ^19^ ^]^ PTBP1 is highly expressed in several tumors and associated with tumorigenesis.^[^ ^55^ ^]^ PTBP1 is an RNA‐binding protein that regulates multiple aspects of the messenger RNA life cycle, including splicing, 3′‐end processing, localization, stability, and translation. It promotes tumor growth in a variety of cancers.^[^ ^56^ ^]^ PTBP1 is highly expressed in breast cancer and its expression is associated with a poor prognosis in patients.^[^ ^57^, ^58^ ^]^ A recent study has shown that PTBP1 can bind to *FOXO1* mRNA^[^ ^43^ ^]^ and XPO1 can bind to PTBP1.^[^ ^42^ ^]^ Our recent study revealed that FOXO1 is highly expressed in BLBC and promotes tumor growth by increasing KLF5 transcription.^[^ ^40^ ^]^ This study revealed that XPO1 can synergistically promote the nuclear export of *FOXO1* mRNA by interacting with PTBP1, ultimately leading to increased transcription of KLF5 (Figure 5).

In summary, our study revealed a novel functional and regulatory mechanisms of KLF5 in BLBC. On the one hand, KLF5 promotes the high expression of XPO1, which in turn increases the nuclear export of RB1. On the other hand, KLF5 increases the expression of *Cyclin D1* and increases the phosphorylation of RB1. Furthermore, we found that XPO1 can bind with PTBP1 to transport *FOXO1* mRNA out of the nucleus and increase the expression of KLF5. Thus, XPO1 may be a potential therapeutic target in BLBC. The combination of XPO1 inhibitors and CDK4/6 inhibitors in BLBC may deserve testing in clinical trials in the future.

## 4 Experimental Section

### Cell Culture and Transfection

BLBC cell lines, HCC1806 and SUM149PT, were purchased from American Type Culture Collection (Manassas, VA, USA) and validated via short tandem repeat analysis, and the cells were cultured in Rose well Park Memorial Institute‐1640 (RPMI‐1640) or DMEM/F12 medium (Gibco, USA) containing 5% fetal bovine serum (FBS, Gibco, USA). HEK293T cells were cultured in Dulbecco's modified Eagle's medium (Gibco, USA) containing 5% FBS. All cells were maintained in an incubator with 5% CO_2_ at 37 °C. All plasmids and siRNA were diluted in Opti‐MEM (Gibco, USA) and transfected into the tumor cell lines using Lipofectamine 2000 (Invitrogen) according to the manufacturer's recommended protocol. Plasmids were transfected into HEK293T cells using 1 µg plasmid: 3 µL PEI (Polyethyleneimine), and the cells were collected after transfection for 48 h. All oligo's information can be queried in Table S1, Supporting Information.

### Co‐Immunoprecipitation and Western Blotting

Cells were washed with PBS and lysed in ice‐cold lysis buffer (IP lysis buffer: 150 mM NaCl, 2 mM ETDA pH 8.0, 50 mM Tris‐HCl pH 7.4, 0.2% NP‐40) for 30 min with a protease inhibitor (MCE, HY‐K0010). Cytoplasmic and nuclear proteins were collected by using nuclear and cytoplasmic protein extraction kit (Beyotime) according to the manufacturer's instructions. Cell lysates were centrifuged at 14, 000 g for 30 min. Protein quantification analysis was performed using a BCA protein assay kit (Abbkine, KTD3001). Cell lysates were incubated with the indicated antibodies overnight at 4 °C, followed by incubation with protein A/G beads (MCE, HY‐K0202), Anti‐Flag beads (MCE, HY‐K0207) for 3–6 h at 4 °C. The beads were washed with cell lysis buffer two to four times. Finally, the beads were boiled in 2×SDS buffer for 10 min at 98 °C. The samples were separated by SDS‐PAGE, transferred onto polyvinylidene fluoride (PVDF) membranes, and blocked with 5% non‐fat dried milk for 1 h at room temperature (RT). The membranes were then incubated with the indicated primary antibody at 4 °C overnight, the corresponding HRP‐conjugated secondary antibody (1:5000) for 1 h at RT, and then detected with a chemiluminescent HRP substrate (Proteintech, SA00001). All antibody information is listed in Table S2, Supporting Information.

### Luciferase Reporter Assays

The DNA fragments were amplified using HCC1806 cell genomic DNA as PCR template and then cloned into pGL3‐Basic vector, respectively. Plasmids were confirmed by Sanger sequencing. HEK293T cells were seeded in 24‐plates and transfected with pCDH‐3×Flag‐KLF5 and pGL3 luciferase reporter plasmids (both 300 ng well^−1^) together with pCMV‐Renilla control (10 ng well^−1^). After transfection for 48 h, cell lysates were collected and the luciferase activities were detected by using the dual‐luciferase reporter assay system (Promega). Primer information for plasmid construction is listed in Table S3, Supporting Information.

### Chromatin Immunoprecipitation

2 × 10^7^ cells were fixed with 1% formaldehyde (Sigma) for 15 min at RT. Crosslinking was quenched by the addition of glycine to a final concentration of 125 mM for 5 min at RT, followed by washing with cold 1×PBS twice. The cells were centrifuged at 750 g for 5 min at 4 °C. Subsequently, the cell pellet was resuspended in 300 µL of ChIP lysis buffer (50 mM Tris‐HCl pH 8.0, 1% SDS, 10 mM EDTA, and 1×PIC). Chromatin DNA was sheared to 200–1, 000 bp average in size through sonication. After centrifugation at 12, 000 g for 10 min at 4 °C, the supernatant was divided into two parts and each of them was diluted in 1.35 mL of dilution buffer (1% Triton X‐100, 2 mM EDTA, 150 mM NaCl, 20 mM Tris‐HCl pH 8.0, and 1×PIC) and immunoprecipitated with specific antibody or IgG overnight at 4 °C, followed by incubation with protein A/G magnetic beads (MCE, HY‐K0202‐1) for an additional 4 h. The beads were sequentially washed for 10 min at 4 °C with TSE1 buffer (20 mM Tris‐HCl pH 8.0, 150 mM NaCl, 2 mM EDTA, 0.1% SDS, 1% Triton X‐100, and 1×PIC), 10 min for TSE2 buffer (20 mM Tris‐HCl pH 8.0, 400 mM NaCl, 0.1% SDS, 1% Triton X‐100, 2 mM EDTA, and 1×PIC), and 1 min for TE buffer (20 mM Tris‐HCl pH 7.4, and 1 mM EDTA). Then the reverse crosslinking was carried out by adding 300 µL TE/SDS (0.5% SDS in TE) and incubated at 65 °C overnight, followed by supplemented with 3 µL of proteinase K (20 mg mL^−1^) at 55 °C for 2 h, 2 µL of RNase A (10 mg mL^−1^) at 37 °C for 30 min. DNA was purified by phenol/chloroform extraction and ethanol precipitation. The pellets were dissolved in 100 µL of ddH_2_O for RT‐qPCR or subjected to high throughput sequencing. PCR primers are listed in Table S4, Supporting Information.

### Immunohistochemistry

The ethics information is as follows: The BLBC specimens’ IHC was approved by Ethics Committee of Henan Provincial People's Hospital (#2020–205).It is consistent with our previously published work.^[^ ^31^ ^]^ Briefly, the tissue samples were fixed with 4% buffered formaldehyde for 48 h at room temperature and embedded in paraffin. Then the paraffin‐embedded tissue sections at 5–8 µm thickness were transferred onto glass slides. The slides were deparaffinized, rehydrated, and pressure cooker heated for 2.5 min in EDTA for antigen retrieval. Endogenous peroxidase activity was inactivated by adding an endogenous peroxidase blocker (OriGene, Beijing, China) for 15 min at room temperature. Slides were incubated overnight at 4 °C with the KLF5 (Proteintech, 66850‐1‐PBS) and XPO1(Proteintech, 66763‐1‐PBS) antibodies. Next, the slides were washed three times with 1×PBS and incubated with secondary antibody (OriGene, Beijing, China) at room temperature for 20 min, DAB concentrate chromogenic solution (1:200 dilution of concentrated DAB chromogenic solution), counterstained with 0.5% hematoxylin, dehydrated with graded concentrations of ethanol for 3 min each (70%‐95%‐100%), and finally stained with dimethyl benzene immune‐stained slides were evaluated by light microscopy. Antibody information is listed in Table S2, Supporting Information.

### Quantitative Reverse Transcription PCR and RNA Sequencing

TRIzol reagent (15596–026, Invitrogen) was used to extract total RNA. HiScript II QRT SuperMix (Vazyme, R223‐01) was used to perform reverse transcription. Quantitative PCR was performed using the SYBR Green Select Master Mix system (Vazyme, Q71202). RNA sequencing was performed and analyzed by LCbio (Hangzhou, China). Primers used for RT‐qPCR are listed in Table S5, Supporting Information.

### Colony Formation and Cell Proliferation Assays

Briefly, 1, 000 cells were seeded per well on a 6‐well plate. Medium with 1 µg mL^−1^ puromycin was changed every 3 days. After about 15 days, the cells were stained with 0.5% crystal violet for 20 min at room temperature. After washing with 1×PBS, the colonies were photographed. Cell proliferation was assayed with a CCK‐8 kit (Melun Bio, MA0218) to measure cell proliferation and viability.

### Cell Cycle Analysis

The cell cycle was analyzed using propidium iodide (PI) staining. HCC1806 and SUM149PT cells were collected and fixed with 75% ethanol overnight. Then, 100 µg mL^−1^ RNase solution was added, and the cells were stained with PI for 30 min. The DNA content was subsequently analyzed by flow cytometry using the Sony ID7000 Flow Cytometer or NovoCyte Advanteon Flow Cytometer.

### Bioinformatics

The binding sites of KLF5 at the TSS of *XPO1* were predicted by JASPAR database (https://jaspar.elixir.no/). The expression of KLF5 and XPO1 in BLBC was analyzed using the GEPIA (http://gepia.cancer‐pku.cn/) and UALCAN (https://ualcan.path.uab.edu/). Correlative expression analysis of KLF5 and XPO1 in the clinic used the Bio‐Portal database (https://www.cbioportal.org/). KPT‐330 and Palbociclib combination effects were analyzed using the Online SynergyFinder web application 3.0(https://synergyfinder.fimm.fi).

### Tumorigenesis in Nude Mice

HCC1806 cells with stable KLF5 and XPO1 knockdown and/or XPO1 overexpression were prepared by lentiviral infection and selected using puromycin, respectively. Knockdown of KLF5/XPO1 and overexpression of XPO1 were confirmed by WB. Twenty‐five female nude mice (6‐week‐old) were randomly distributed into five groups (shNC+pCDH, shKLF5+pCDH, shKLF5+XPO1‐3×Flag, shXPO1+pCDH and shNC+XPO1‐3×Flag; each group contained five mice). HCC1806 cells (1 × 10^6^) were injected into the mammary fat pads of the mice. The length and width of the tumor were measured every 3 days using a Vernier calipers. The tumor volumes were calculated as follows: tumor volume (mm^3^) = length×width^2^. The mice were sacrificed on day 24, and the tumors were harvested and weighed. The animal experiment was approved (KMMU20240888) by Animal Ethics Review Committee of Kunming Medical University.

### Oral Administration of Palbociclib and Selinexor to Nude Mice with Tumors

HCC1806 cells (1 × 10^6^) were injected into both the left and right mammary fat pads of twelve female nude mice (6‐7 weeks‐old). After 5 days, the tumor volume and weight of mice were measured, and mice were randomly distributed into four groups. The mice were then treated with Palbociclib (TSBiochem, 120 208) or Selinexor (TSBiochem, T6106) by oral administration every 2 days. The tumor volume and mouse weight were measured every other day. The mice were sacrificed on day 21, and the tumors were harvested and weighed. Palbociclib and Selinexor was prepared by adding each of the following solvents in sequential order; 10% DMSO, 40% PEG300, 5% Tween‐80, and 45% saline. The drug solution was freshly prepared to avoid freeze thawing that could cause drug precipitation.

### Statistical Analysis

Student's *t*‐test (2‐tailed) was used to compare differences between two groups. One‐way ANOVA comparison test was used to analyze the differences among multiple groups. Data are presented as means± standard deviation (*SD*). *p* values of <0.05 were considered significant. All statistical data were calculated using the GraphPad Prism 8 (GraphPad Software Inc., La Jolla, CA, USA).

## Conflict of Interest

The authors declare no conflict of interest.

## Author Contributions

Y.T., R.L., J.Z., Q.H., and C.P. contributed equally to this work. C.C., D.J., and J.Y. designed and supervised the study. Y.T., R.L., and J.Z. performed most of the experiments and analyses. Q.H., C.P., Z.Z., J.S., and F.L. participated in some experiments. C.P. and Y.T. performed and analyzed clinical and experimental IHC data. Q.H., Y.T., C.P., and J.Z. participated in discussion. Y.S. provided tissue microarrays. Y.T., D.J., and C.C. draft the manuscript. All authors discussed and finalized this paper.

## Supporting information



Supporting Information

## Data Availability

Data sharing is not applicable to this article as no new data were created or analyzed in this study.
