# Supplementary material for: Positive Feedback Regulation between KLF5 and XPO1 Promotes Cell Cycle Progression of Basal like Breast Cancer
Source: Adv Sci (Weinh). 2025 Jan 30;12(16):2412096. doi: 10.1002/advs.202412096 (PMC12021099; doi:10.1002/advs.202412096)
Supplement: Supplementary file 1 — Supporting Information [file ADVS-12-2412096-s001.docx]

**Supplementary Materials for:**

**Positive Feedback Regulation between KLF5 and XPO1 Promotes Cell Cycle Progression of Basal Like Breast Cancer**

Yu Tang^1#^, Rui Liu^1#^, Jing Zhu^2#^, Qian He^2#^, Chenglong Pan^3#^, Zhongmei Zhou^4^, Jian Sun^1^, Fubing Li^2^, Longlong Zhang^2^, Yujie Shi^5^, Jing Yao^6, 7*^, Dewei Jiang^8*^, Ceshi Chen^1,2*^

^1^Yunnan Key Laboratory of Breast Cancer Precision Medicine, Yunnan Cancer Hospital, The Third Affiliated Hospital of Kunming Medical University, Peking University Cancer Hospital Yunnan, Kunming, 650118, China.

^2^Yunnan Key Laboratory of Breast Cancer Precision Medicine, Academy of Biomedical Engineering, Kunming Medical University, Kunming, 650000, China.

^3^Department of Pathology, The First Affiliated Hospital of Kunming Medical University, Kunming, 650032, China.

^4^School of Continuing Education, Kunming Medical University, Kunming, 650021, China.

^5^Department of Pathology, Henan Provincial People's Hospital, Zhengzhou University, Zhengzhou, 450003, China.

^6^Cancer Center, Union Hospital, Tongji Medical College, Huazhong University of Science and Technology, Wuhan 430022, China.

^7^Institute of Radiation Oncology, Union Hospital, Tongji Medical College, Huazhong University of Science and Technology, Wuhan 430022, China.

^8^Key Laboratory of Animal Models and Human Disease Mechanisms of Yunnan Province, Kunming Institute of Zoology, Chinese Academy of Sciences, Kunming, 650201, China.

^#^ These authors contribute equally

**Corresponding to [chenc@kmmu.edu.cn](mailto:chenc@kmmu.edu.cn), jiangdewei@mail.kiz.ac.cn, or [2007XH0839@hust.edu.cn](mailto:2007XH0839@hust.edu.cn)**

**This file includes:**

** Supplementary Figures S1-S5 (with legends)**

** Supplementary Experimental Section**

** Supplementary Table S1-S5**

**Supplementary Figures**


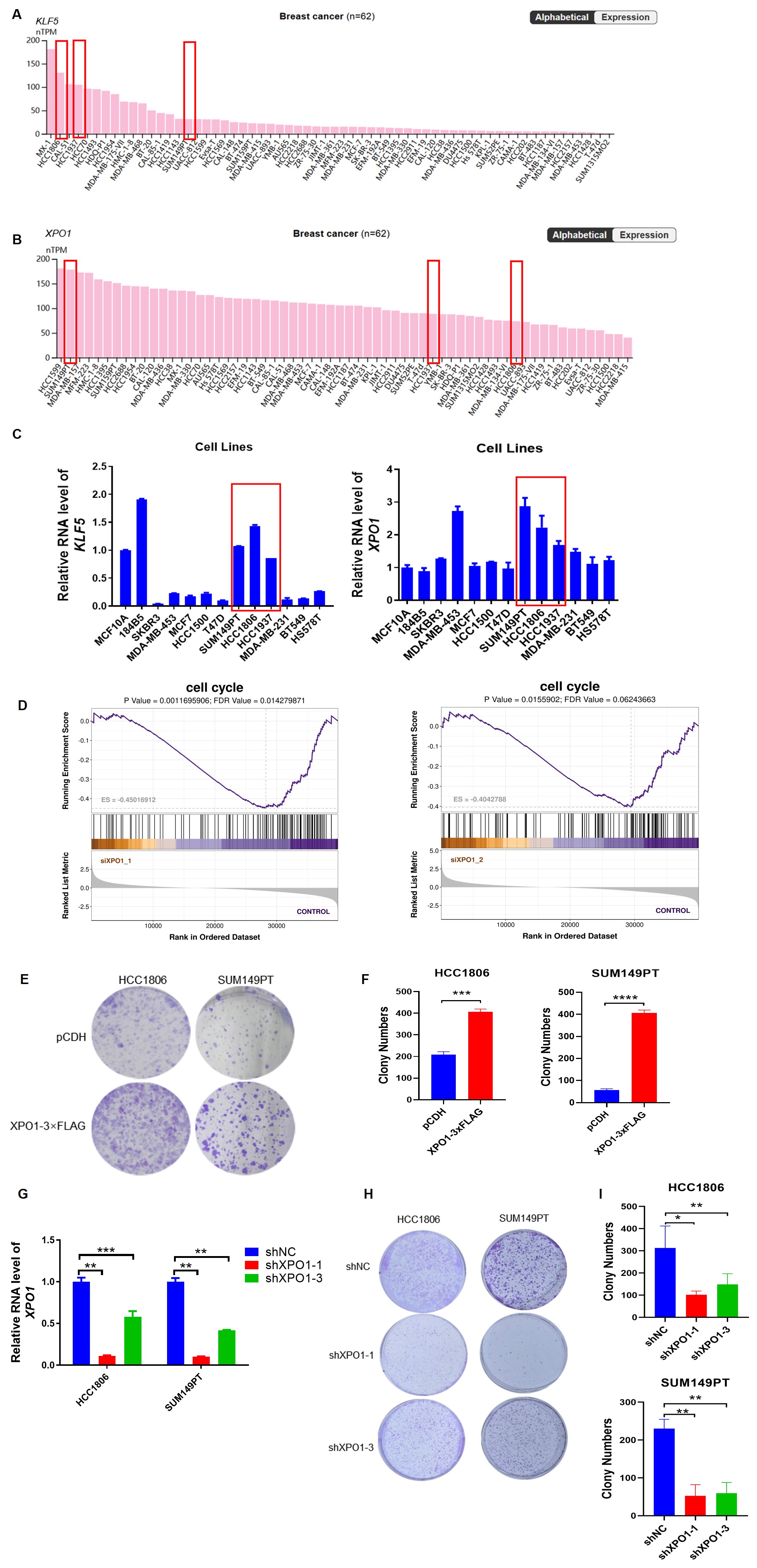


**Supplementary Figure S1. XPO1 promotes BLBC cell proliferation.**

A) The expression of *KLF5* in breast cancer cell lines was queried using The Human Protein Atlas database. B) The expression of *XPO1* in breast cancer cell lines was queried using The Human Protein Atlas database. C) The mRNA expression of *KLF5* and *XPO1* was detected by RT-qPCR. Extracting the total RNA inside the normal breast epithelial cells and breast cancer cell lines (n=3). D) The differential genes were analyzed by GSEA, and the cell cycle is enriched. After XPO1 was knocked down in HCC1806 cells, the RNA-seq was performed. E) XPO1 was overexpressed in BLBC cells, and the proliferation of BLBC cells was detected by colony formation assay (n=3). F) Quotative results of panel E. ****p*<0.001, *****p*<0.0001. G) XPO1 knocked down in BLBC cells, *XPO1* mRNA expression was detected by RT-qPCR (n=3). ***p*<0.01, ****p*<0.001. H) XPO1 was knocked down in BLBC cells, and the proliferation of BLBC cells was detected by colony formation assay (n=3). I) Quotative results of panel H. **p*<0.05, ***p*<0.01.


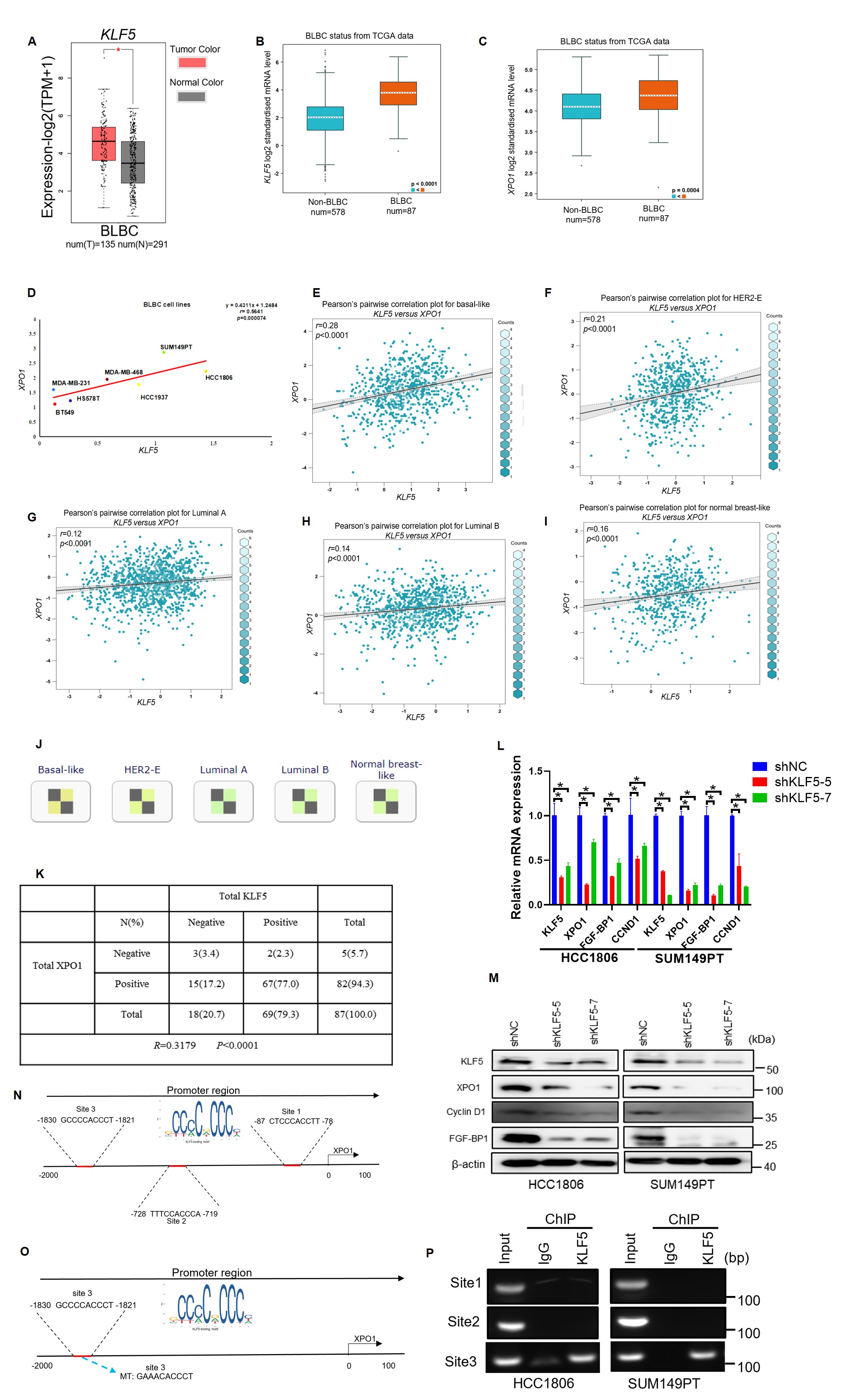


**Supplementary Figure S2. KLF5 promotes *XPO1* gene transcription and cell proliferation through XPO1 in BLBC.**

A) The expression of *KLF5* in BLBC clinical samples was detected using the GEPIA online database. **p*<0.05. B) The expression of *KLF5* in different types of breast cancer was detected, using the bc-GenExMiner v4.5 online database. C) The expression of *XPO1* in different types of breast cancer was detected, using the bc-GenExMiner v4.5 online database. D) *KLF5* and *XPO1* mRNA expression in BLBC cells was detected by RT-qPCR and the correlation was analyzed (n=3). E) The expression of *KLF5* and *XPO1* positively correlated in BLBC patients. Query using TCGA online database. F) The expression of *KLF5* and *XPO1* was positively correlated in HER2-E patients. Query using the TCGA online database. G) The expression of *KLF5* and *XPO1* was positively correlated in Luminal A patients. Query using the TCGA online database. H) The expression of *KLF5* and *XPO1* was positively correlated in Luminal B patients. Query using the TCGA online database. I) The expression of *KLF5* and *XPO1* was positively correlated in normal breast-like patients. Query using the TCGA online database. J) Among the different breast cancer patients analyzed, *KLF5* and *XPO1* showed the strongest association in BLBC. K) There was a positive correlation between KLF5 and XPO1 expression in 87 samples of BLBC patients. The correlation between IHC staining results of clinical BLBC patients' samples was analyzed (n=87). L) After KLF5 knockdown in BLBC cells, the mRNA expression changes of XPO1, FGFBP1 and CCND1 genes were detected by qPCR (n=3). **p*<0.05. M) After KLF5 knockdown in BLBC cells, the expression levels of XPO1, FGFBP1 and Cyclin D1 proteins were detected by WB. N) KLF5 has three potential binding sites in the XPO1 promoter region, which were predicted by JASPER online tool. O) Diagram of the mutation site 3 in the XPO1 promoter region. P) KLF5 bound to XPO1 promoter region's site 3, but not site1 and site2, detected by ChIP-PCR (n=3).


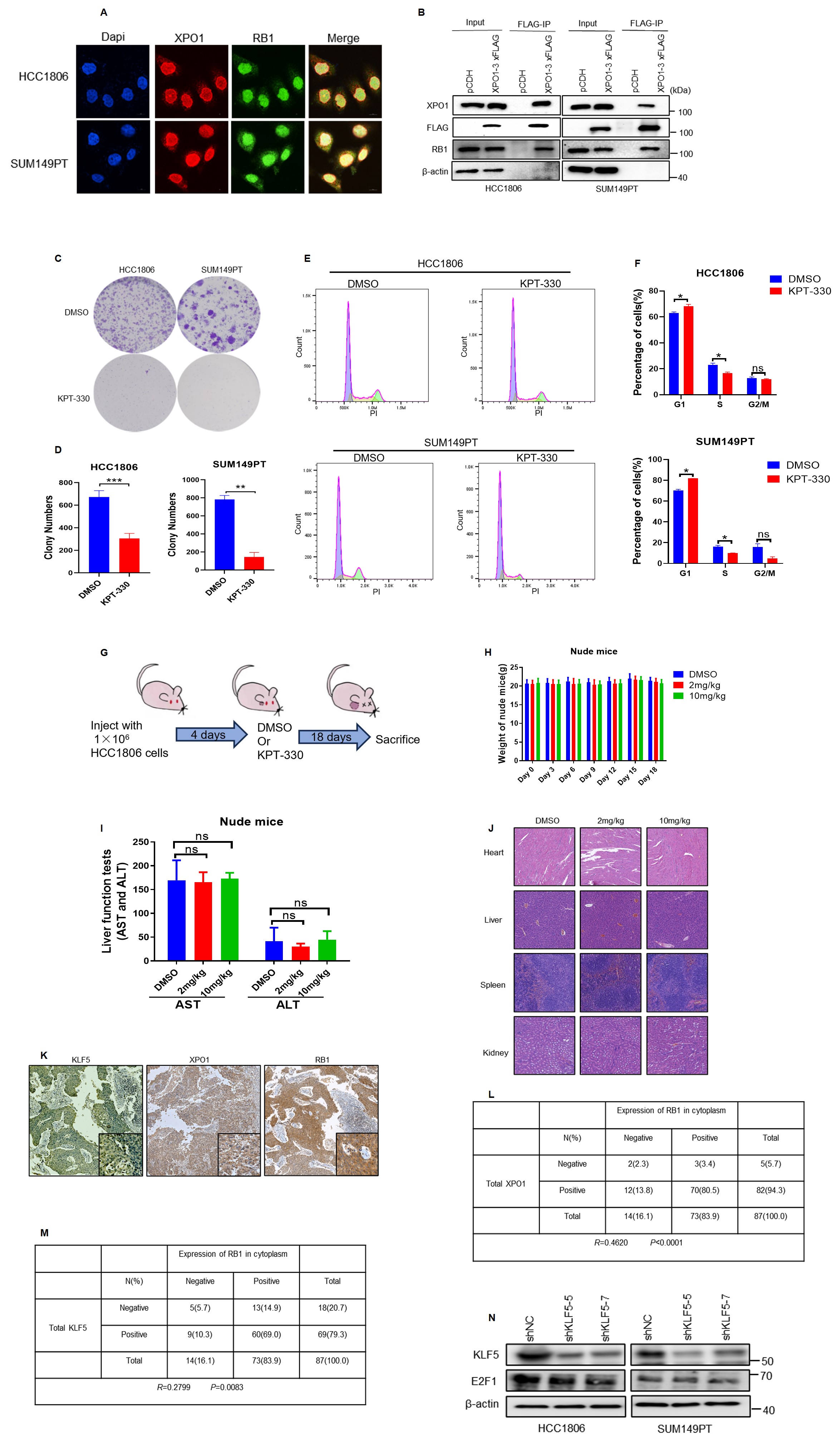


**Supplementary Figure S3. XPO1 promotes BLBC cell proliferation by transporting RB1 protein out of the nucleus.**

A) The expression of XPO1 and RB1 proteins in the nucleus or cytoplasm of BLBC cells was detected by immunofluorescence. B) Exogenous Co-IP experiments demonstrated the presence of binding between XPO1 and RB1. IP experiments were performed by infection with XPO1-3×FLAG lentivirus followed by FLAG magnetic beads. C) KPT-330 can inhibit the proliferation of BLBC cells. The results of the clone formation assay were analyzed (n=3). D) Quotative results of panel B. ***p*<0.01, ****p*<0.001. E) KPT-330 treats BLBC cells for 48h can arrest the cell cycle in the G1 phase, it was measured by flow cytometry (n=3). F) Quotative results of panel D. **p*<0.05, n. s, not significant. G) HCC1806 cells (1×10^6^ cells/site) were injected into the fourth pair of nipple fat pad of nude mice, and different concentrations of KPT-330 were given 4 days later. The nude mice were killed 18 days after treatment (n=7 per group). H) There were no significant changes in body weight during the KPT-330 treatment of nude mice (n=7 per group). I) Liver function in nude mice has no significant change after treatment with KPT-330 (n=7 per group). n. s, not significant. J) The heart, liver, spleen and kidney of the nude mice were collected and stained with HE to detect the toxic and side effects of the drug after nude mice were treated with KPT-330. K) The expression of KLF5, XPO1 and cytoplasmic RB1 proteins in BLBC patient tissue samples, as assessed by immunohistochemical (IHC) staining. The images are shown at 5× and 40× magnification (n=87). L) There was a positive correlation between XPO1 and cytoplasmic RB1 expression in 87 samples of BLBC patients. The correlation between IHC staining results of clinical BLBC patients' samples was analyzed (n=87). M) There was a positive correlation between KLF5 and cytoplasmic RB1 expression in 87 samples of BLBC patients. The correlation between IHC staining results of clinical BLBC patients' samples was analyzed (n=87). N) KLF5 knockdown in BLBC does not cause changes in E2F1 expression， as detected by WB.


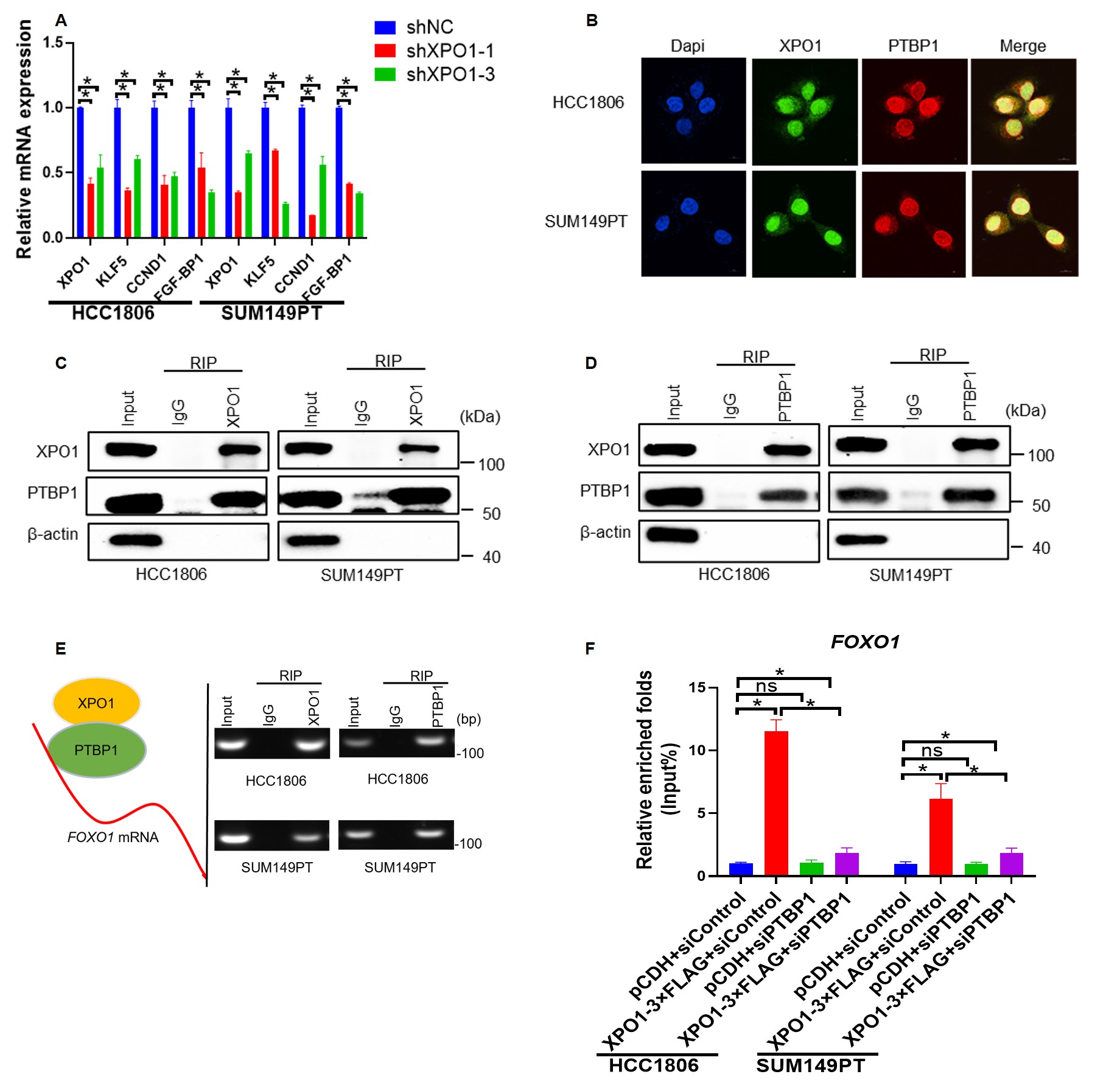


**Supplementary Figure S4. XPO1 promotes KLF5 expression by binding to PTBP1 and *FOXO1* mRNA nuclear export in BLBC cells.**

A) After XPO1 knockdown in BLBC cells, the mRNA expression of KLF5 and its downstream target genes was detected by qPCR (n=3). **p*<0.05. B) The expression of XPO1 and PTBP1 in the nucleus and cytoplasm was detected by immunofluorescence assay. C) XPO1 bound to thePTBP1 protein in BLBC cells. RIP was performed with the anti-XPO1 Ab. WB examined proteins. D) PTBP1 bound to the XPO1 protein in BLBC cells. RIP was performed with the anti-PTBP1 Ab. WB examined proteins. E) XPO1 and PTBP1 bound to the *FOXO1* mRNA. RIP-PCR was used to detect the presence of *FOXO1* mRNA after RIP. F) XPO1-3×FLAG was overexpressed in BLBC cells by lentivirus, then PTBP1 was knockdown by siRNA in BLBC cells, and the mRNA enrichment rate of FOXO1 was detected by FLAG-RIP qPCR assay (n=3). **p*<0.05, n. s, not significant.


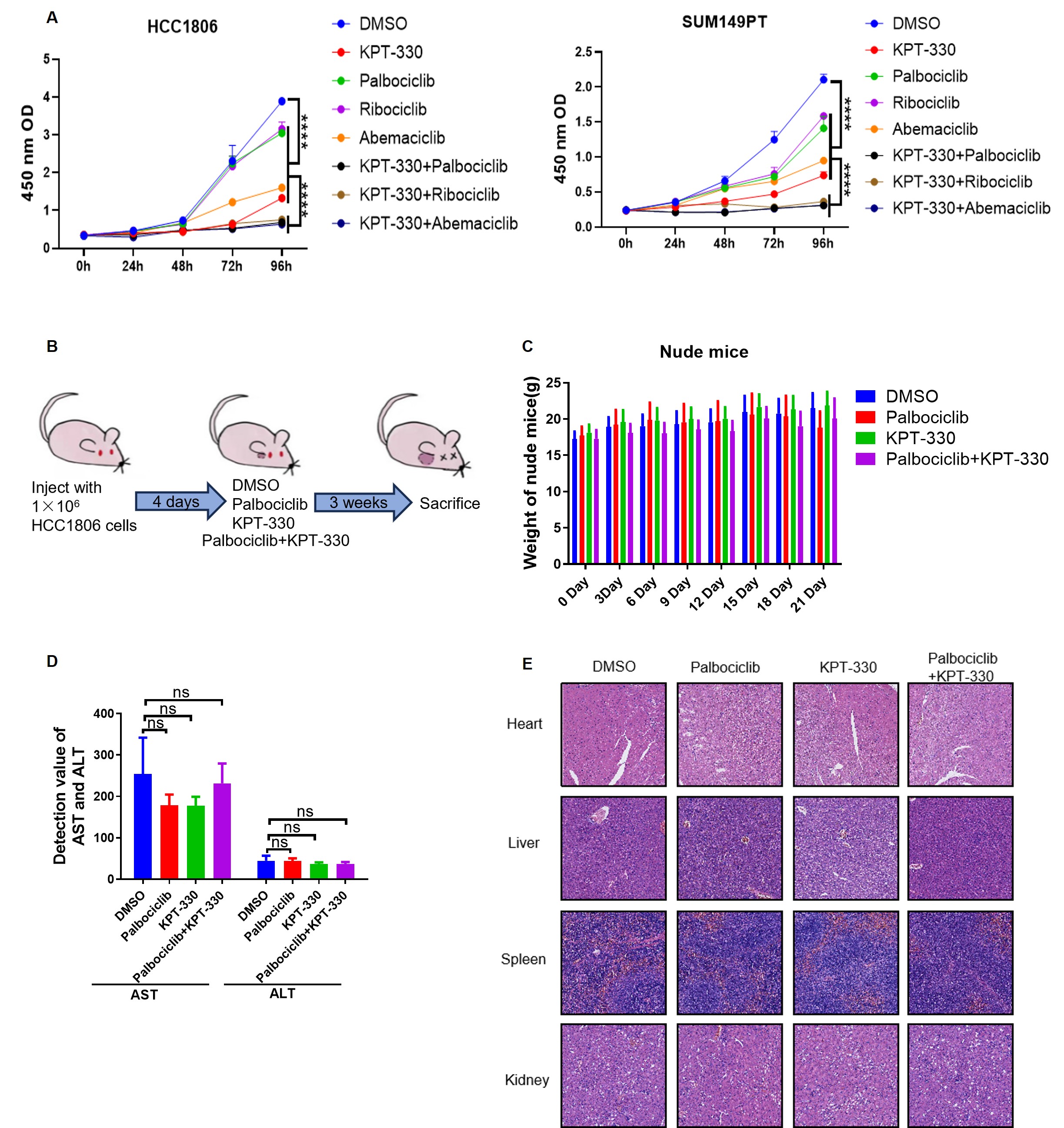


**Supplementary Figure S5. CDK4/6 inhibitor Palbociclib, in combination with XPO1 inhibitor KPT-330, shows an additive therapeutic effect on BLBC.**

A) BLBC cells were treated with different CDK4/6 inhibitors in combination with KPT-330, and then the proliferation activity of BLBC cells was detected by CCK-8 assay. ****p<0.0001. B) The fourth pair of nude mice were injected with HCC1806 cells (1×10^6^ cells/site) into the nipple fat pad. Four days later, the mice were given different drugs, and drug treatment was given for 21 days (n=5 per group). C) There were no significant changes in the body weight of nude mice during the treatment of BLBC with Palbociclib in combination with KPT-330 (n=5 per group). D) The liver function indexes of nude mice showed no obvious abnormality after different treatments were given to nude mice (n=5 per group). n. s, not significant. E) The heart, liver, spleen, and kidney of nude mice were collected, and HE staining was performed to detect the drug toxicity; there was no significant morphological change in the organ after drug treatment.

**Supplementary Experimental Section**

**Immunofluorescence assay**

Cells on glass coverslips were fixed with 4% para formaldehyde and permeabilized with 0.2% Triton X-100 in PBS. Samples were blocked in 5% BSA in the presence of 0.1% Triton X-100 and stained with the appropriate primary and secondary antibodies coupled to Alexa Fluor 488, 594 (AB Clonal). The primary antibodies were diluted in phosphate buffer saline with 0.1% Tween-20 (PBST) containing 5% BSA and incubated with cells overnight at 4 °C. After washing, secondary antibodies were diluted in PBST containing 5% BSA and incubated with cells at room temperature for 1h. Then cells were washed twice and incubated with DAPI (HY-D0814, MCE, Shanghai, China) at room temperature for 5 min. After twice washing, cells were mounted with anti-fade mounting medium. Confocal images were captured on a Nikon A1MP+ Microscope with a ×40 oil objective.

**RNA immunoprecipitation (RIP）**

Cells were washed twice with PBS, collected and then the pellet was resuspended in IP lysis buffer (150 mM KCl, 25 mM Tris (pH 7.4), 5 mM EDTA, 0.5 mM DTT, 0.5% NP40, 1× protease inhibitor, 1 U/μl RNase inhibitor). The lysate was harvested by centrifugation at 12 000 g for 10 min after incubation for 30 min. Antibodies and 40 μl of protein G beads (Invitrogen, USA) were added into the lysate followed by incubation overnight at 4°C. After washed three times with wash buffer (150 mM KCl, 25 mM Tris (pH 7.4), 5 mM EDTA, 0.5 mM DTT, 0.5% NP40), co-precipitated RNAs were extracted by Trizol reagent, ethanol-precipitated with glycogen (Invitrogen, USA). The enrichment of RNAs was normalized to IgG.

**Supplementary Tables**

**Supplementary Table S1. Oligo sequence**

| **Oligo** | **Sequence (5’-3’)** |
| --- | --- |
| **shXPO1-1** | F: CGGgctcaagaagtactgacacatCTCGAGatgtgtcagtacttcttgagcTTTTTG  R: AATTCAAAAAgctcaagaagtactgacacatTCGAGatgtgtcagtacttcttgagc |
| **shXPO1-3** | F: CCGGattcgacttgcgtactcaaatCTCGAGatttgagtacgcaagtcgaatTTTTTG  R: AATTCAAAAAattcgacttgcgtactcaaatCTCGAGatttgagtacgcaagtcgaat |
| **siControl** | UUCUCCGAACGUGUCACGUTT  ACGUGACACGUUCGGAGAATT |
| **siRB1-2** | UGUAAGAUCUCCAAAGAAATT  UUUCUUUGGAGAUCUUACATT |
| **siRB1-3** | GAACAGGAGUGCACGGAUATT  UAUCCGUGCACUCCUGUUCTT |
| **siKLF5-1** | CGAUUACCCUGGUUGCACATT  UGUGCAACCAGGGUAAUCGTT |
| **siKLF5-2** | AAGCUCACCUGAGGACUCATT  UGAGUCCUCAGGUGAGCUUTT |
| **siPTBP1-1** | AGGAAAACGGAAAGAAUGCGATT  GCAUUCUUUCCGUUUUCCUGATT |
| **siPTBP1-2** | AAAGAAUGCGAAACAUCUCCATT  GAGAUGUUUCGCAUUCUUUCCTT |

**Supplementary Table S2. Antibody Information**

| **Antibodies** | **SOURCE** | **IDENTIFIER** |
| --- | --- | --- |
| **anti-KLF5** | Abcam | Cat# AB137676 |
| **anti-FGFBP1** | Abcam | Cat# AB215353 |
| **anti-cyclin D1** | Cell Signaling Technology | Cat# 55506S |
| **anti-KLF5（E5K6H）** | Cell Signaling Technology | Cat# 40674S |
| **anti-KLF5（D7S3F）** | Cell Signaling Technology | Cat# 51586S |
| **anti-Cyclin D1** | Cell Signaling Technology | Cat# 2922S |
| **anti-PTBP1** | Cell Signaling Technology | Cat# 57246S |
| **anti-CRM1** | Cell Signaling Technology | Cat# 46249S |
| **anti-FOXO1** | Cell Signaling Technology | Cat# 2880S |
| **anti-p-Rb** | Cell Signaling Technology | Cat# 8516S |
| **anti-FOXO1** | MCE | Cat# HY-P80676 |
| **anti-YB1** | MCE | Cat# HY-P80375 |
| **anti-h/mKLF5** | RD | Cat# AF3758 |
| **anti-DDDDK-tag** | MBL | Cat# M185-3L |
| **anti-GAPDH** | Proteintech | Cat# 60004-1-Ig |
| **anti-KLF5** | Proteintech | Cat# 66850-1-Ig |
| **anti-Beta Actin** | Proteintech | Cat# 66009-1-Ig |
| **anti-RB1** | Proteintech | Cat# 65721-1-Ig |
| **anti-CRM1** | Proteintech | Cat# 66763-1-Ig |
| **anti-Histone 3** | Proteintech | Cat# 17168-1-AP |
| **HRP-conjugated Affinipure Goat Anti-Mouse IgG(H+L)** | Proteintech | Cat# SA00001-1 |
| **Rabbit IgG control Rabbit polyAb** | Proteintech | Cat# 30000-0-AP |
| **HRP-conjugated Affinipure Rabbit Anti-Goat IgG(H+L)** | Proteintech | Cat# SA00001-4 |
| **Goat-IgG Rabbit PolyAb** | Proteintech | Cat# 10285-1-AP |

**Supplementary Table S3. Plasmid primers**

| **Plasmid primer name** | **Sequence (5’-3’)** |
| --- | --- |
| **XPO1-pCDH-3×FLAG** | F: agagaattcggatccATGCCAGCAATTATGACAATGTTAG  R: cttccatggctcgagTTAATCACACATTTCTTCTGGAATCTC |
| **pXPO1-PGL3-Basic** | F: atttctctatcgataggtaccAATTATTGTATTTTTGGTAGAGACC  R: cagtaccggaatgccaagcttCCCGCAGAGTGGGGGA |
| **Site1-PGL3-Basic** | F: atttctctatcgataggtaccAATTATTGTATTTTTGGTAGAGACC  R: cagtaccggaatgccaagcttCAACAATAAAAATAATACAAAATAA |
| **Site2-PGL3-Basic** | F: atttctctatcgataggtaccAATTATTGTATTTTTGGTAGAGACC  R: agtaccggaatgccaagcttTCTCTCCAGGCTGGAATTTGGCTG |
| **Site3-PGL3-Basic** | F: atttctctatcgataggtaccAATTATTGTATTTTTGGTAGAGACC  R: cagtaccggaatgccaagcttGAAGCCGCGTGGAGGATT |

**Supplementary Table S4. ChIP-PCR primers**

| **ChIP-PCR primer name** | **Sequence (5’-3’)** |  |
| --- | --- | --- |
| **Site1** | F: CAATGCTGGGATTACAGGC  R: ACGGGACTCGTTCTCAAA |  |
| **Site2** | F: GTGCTTTGAGGTTAGCCATAG  R: GCCACCAAGAGGTAGATAAATG |  |
| **Site3** | F: AAGCAAAGAGAACGCTGC  R: TGTCAATCAAGAGCAGCCC | |

**Supplementary Table S5. RT-qPCR Primers**

| **RT-qPCR Primer name** | **Sequence (5’-3’)** |
| --- | --- |
| ***KLF5*** | F: ACACCAGACCGCAGCTCCA  R: TCCATTGCTGCTGTCTGATTTGTAG |
| ***XPO1*** | F: ATCTGACCCAACTTGTGTAGAGA  R: TGGTCCTACTTGCTCCAACAAT |
| ***FOXO1*** | F: AAGAGCGTGCCCTACTTCAA  R: CTGTTGTTGTCCATGGATGC |
| ***PTBP1*** | F: CACCGCTTCAAGAAACCAGGCT  R: GTTGCTGGAGAAGAGGCTCTTG |
